# Ribonucleicacid interference or small molecule inhibition of *Runx*1 in the border zone prevents cardiac contractile dysfunction following myocardial infarction

**DOI:** 10.1093/cvr/cvad107

**Published:** 2023-07-11

**Authors:** Tamara P Martin, Eilidh A MacDonald, Ashley Bradley, Holly Watson, Priyanka Saxena, Eva A Rog-Zielinska, Anmar Raheem, Simon Fisher, Ali Ali Mohamed Elbassioni, Ohood Almuzaini, Catriona Booth, Morna Campbell, Alexandra Riddell, Pawel Herzyk, Karen Blyth, Colin Nixon, Lorena Zentilin, Colin Berry, Thomas Braun, Mauro Giacca, Martin W McBride, Stuart A Nicklin, Ewan R Cameron, Christopher M Loughrey

**Affiliations:** British Heart Foundation Glasgow Cardiovascular Research Centre, School of Cardiovascular and Metabolic Health, University of Glasgow, University Place, Glasgow G12 8TA, UK; British Heart Foundation Glasgow Cardiovascular Research Centre, School of Cardiovascular and Metabolic Health, University of Glasgow, University Place, Glasgow G12 8TA, UK; British Heart Foundation Glasgow Cardiovascular Research Centre, School of Cardiovascular and Metabolic Health, University of Glasgow, University Place, Glasgow G12 8TA, UK; British Heart Foundation Glasgow Cardiovascular Research Centre, School of Cardiovascular and Metabolic Health, University of Glasgow, University Place, Glasgow G12 8TA, UK; British Heart Foundation Glasgow Cardiovascular Research Centre, School of Cardiovascular and Metabolic Health, University of Glasgow, University Place, Glasgow G12 8TA, UK; Faculty of Medicine, Institute for Experimental Cardiovascular Medicine, University Heart Centre Freiburg/Bad Krozingen, 79110 Freiburg, Germany; British Heart Foundation Glasgow Cardiovascular Research Centre, School of Cardiovascular and Metabolic Health, University of Glasgow, University Place, Glasgow G12 8TA, UK; British Heart Foundation Glasgow Cardiovascular Research Centre, School of Cardiovascular and Metabolic Health, University of Glasgow, University Place, Glasgow G12 8TA, UK; British Heart Foundation Glasgow Cardiovascular Research Centre, School of Cardiovascular and Metabolic Health, University of Glasgow, University Place, Glasgow G12 8TA, UK; Department of Cardiothoracic Surgery, Suez Canal University, 41522 Ismailia, Egypt; British Heart Foundation Glasgow Cardiovascular Research Centre, School of Cardiovascular and Metabolic Health, University of Glasgow, University Place, Glasgow G12 8TA, UK; British Heart Foundation Glasgow Cardiovascular Research Centre, School of Cardiovascular and Metabolic Health, University of Glasgow, University Place, Glasgow G12 8TA, UK; British Heart Foundation Glasgow Cardiovascular Research Centre, School of Cardiovascular and Metabolic Health, University of Glasgow, University Place, Glasgow G12 8TA, UK; British Heart Foundation Glasgow Cardiovascular Research Centre, School of Cardiovascular and Metabolic Health, University of Glasgow, University Place, Glasgow G12 8TA, UK; School of Molecular Biosciences, University of Glasgow, Glasgow G12 8QQ, UK; College of Medical, Veterinary and Life Sciences, Glasgow Polyomics, University of Glasgow, Garscube Campus, Glasgow G61 1BD, UK; School of Cancer Sciences, University of Glasgow, Glasgow G12 0YN, UK; Cancer Research UK Beatson Institute, Garscube Estate, Glasgow G12 0YN, UK; Cancer Research UK Beatson Institute, Garscube Estate, Glasgow G12 0YN, UK; Molecular Medicine Laboratory, International Centre for Genetic Engineering and Biotechnology, 34149 Trieste, Italy; British Heart Foundation Glasgow Cardiovascular Research Centre, School of Cardiovascular and Metabolic Health, University of Glasgow, University Place, Glasgow G12 8TA, UK; Department of Cardiac Development and Remodelling, Max Planck Institute for Heart and Lung Research, 61231 Bad Nauheim, Germany; Molecular Medicine Laboratory, International Centre for Genetic Engineering and Biotechnology, 34149 Trieste, Italy; School of Cardiovascular Medicine and Sciences, King’s College London British Heart Foundation Centre, London WC2R 2LS, UK; British Heart Foundation Glasgow Cardiovascular Research Centre, School of Cardiovascular and Metabolic Health, University of Glasgow, University Place, Glasgow G12 8TA, UK; British Heart Foundation Glasgow Cardiovascular Research Centre, School of Cardiovascular and Metabolic Health, University of Glasgow, University Place, Glasgow G12 8TA, UK; School of Biodiversity, One Health and Veterinary Medicine, University of Glasgow, Glasgow G12 0YN, UK; British Heart Foundation Glasgow Cardiovascular Research Centre, School of Cardiovascular and Metabolic Health, University of Glasgow, University Place, Glasgow G12 8TA, UK

**Keywords:** Cardiomyocytes, Myocardial Infarction, *Runx*1

## Abstract

**Aims:**

Myocardial infarction (MI) is a major cause of death worldwide. Effective treatments are required to improve recovery of cardiac function following MI, with the aim of improving patient outcomes and preventing progression to heart failure. The perfused but hypocontractile region bordering an infarct is functionally distinct from the remote surviving myocardium and is a determinant of adverse remodelling and cardiac contractility. Expression of the transcription factor RUNX1 is increased in the border zone 1-day after MI, suggesting potential for targeted therapeutic intervention.

**Objective:**

This study sought to investigate whether an increase in RUNX1 in the border zone can be therapeutically targeted to preserve contractility following MI.

**Methods and results:**

In this work we demonstrate that *Runx*1 drives reductions in cardiomyocyte contractility, calcium handling, mitochondrial density, and expression of genes important for oxidative phosphorylation. Both tamoxifen-inducible *Runx*1-deficient and essential co-factor common β subunit (*Cbf*β)-deficient cardiomyocyte-specific mouse models demonstrated that antagonizing RUNX1 function preserves the expression of genes important for oxidative phosphorylation following MI. Antagonizing RUNX1 expression via short-hairpin RNA interference preserved contractile function following MI. Equivalent effects were obtained with a small molecule inhibitor (Ro5-3335) that reduces RUNX1 function by blocking its interaction with CBFβ.

**Conclusions:**

Our results confirm the translational potential of RUNX1 as a novel therapeutic target in MI, with wider opportunities for use across a range of cardiac diseases where RUNX1 drives adverse cardiac remodelling.

**Time of primary review: 32 days**

## 1. Introduction

Myocardial infarction (MI) due to acute coronary artery blockage leads to cardiomyocyte death and an injury response culminating in the generation of three regions within the left ventricle (LV). The infarct zone (IZ) is predominantly comprised of fibrillar collagens that maintain the integrity of the myocardium and prevent LV wall rupture. The remote zone (RZ) is located furthest away from the IZ but over time develops several cellular and extracellular matrix changes that impact LV function. The border zone (BZ) surrounds the IZ. Myocardium in the BZ is viable and perfused but hypocontractile. Distinct cellular changes across the three regions are fundamental to the process of adverse cardiac remodelling, which manifests clinically as LV wall thinning, dilation, and reduced contractility as early as 1-day post-MI.^1–6^ Together with neurohumoral activation, adverse cardiac remodelling post-MI can lead to the clinical syndrome of heart failure (HF) with reduced ejection fraction, which despite optimized medical and device therapy is associated with high mortality rates.^7^

Cellular changes that occur in the BZ during the first few days post-MI play a critical role in the progression of LV remodelling, contractility, and patient prognosis during the subsequent weeks and months.^6,8–12^ Many of these BZ changes are conserved between mice and humans and include abnormal sarcoplasmic reticulum (SR)-mediated calcium release^1,2,13–16^ and the downregulation of genes important for mitochondrial oxidative phosphorylation.^17^ The identification of drivers that induce these early changes in the BZ is critical for the development of new therapeutic approaches to prevent the progression of adverse cardiac remodelling and the development of HF following MI.

RUNX1 encodes a DNA-binding α-subunit that partners with a common β subunit (CBFβ) to form a heterodimeric transcription factor that acts as both an activator and repressor of target genes in normal development and disease.^18^ Although RUNX1 has been intensively studied in cancer and haematology, its role in the heart is only just emerging.^19^*Runx*1 expression is increased in cardiomyocytes located within the BZ and IZ region as early as 1-day post-MI and mediates impaired contractility.^20–22^ Whether this increased RUNX1 expression in the BZ can be therapeutically targeted to preserve contractility following MI was unknown.

## 2. Methods

Detailed methods and statistical analysis are presented in the Supplementary material online.

Briefly, *Runx*1^fl/fl^ mice and *Cbf*β^fl/fl^ mice were crossed with mice expressing tamoxifen-inducible Cre recombinase (MerCreMer) under the control of the cardiac-specific αMHC (α-myosin heavy chain) to generate the relevant test and littermate control groups. MI was induced through a thoracotomy and permanent ligation of the left anterior descending coronary artery.

Calcium measurements were performed on cardiomyocytes which were isolated according to region. BZ and remote RZ were loaded with a calcium-sensitive fluorophore (5.0 μmol/L Fura-4F AM, Invitrogen), and perfused during field stimulation (1.0 Hz, 2.0 ms duration, stimulation voltage set to 1.5 times the threshold).

RNA sequencing was performed on BZ and RZ myocardial tissue from control *Runx*1*^fl^*^/*fl*^ and *Runx*1-deficient (*Runx*1^Δ/Δ^) mice pre-MI and at 1-day post-MI and whole LV tissue from control *Cbf*β*^fl^*^/fl^ and *Cbf*β-deficient (*Cbf*β^Δ/Δ^) mice pre-MI and at 7-days post-MI.

Knock down of *Runx*1 was achieved by either adenoviral (Ad), adeno-associated viral (AAV), or a small molecular inhibitor (Ro5-3335) as detailed in the online appendix.

## 3. Results

### 3.1 *Runx*1 impairs BZ Ca^2+^ handling

**Figure 1:**
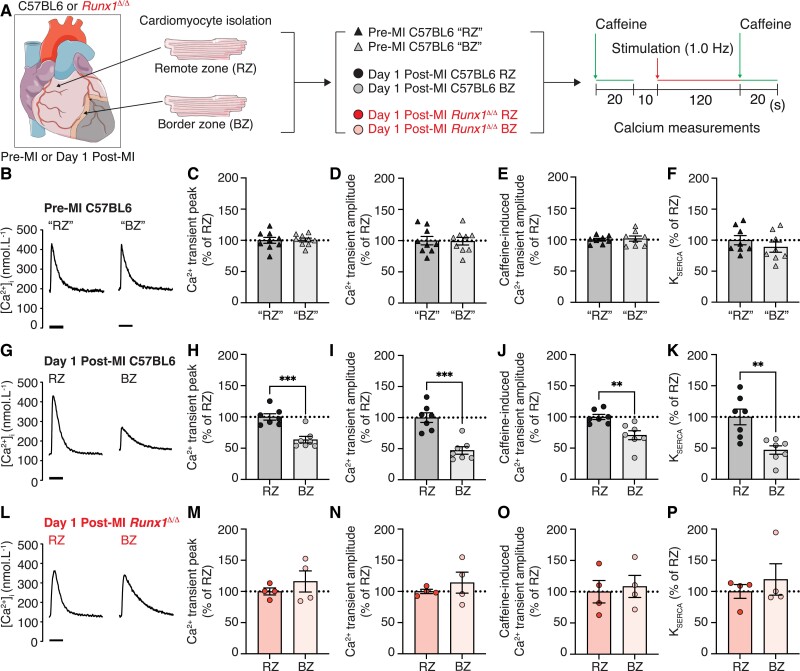
*Runx1* alters BZ Ca^2+^. (*A*) Protocol. (*B*) Typical calcium (Ca^2+^) transients pre-MI in C57BL/6J mice. Mean (*C*) Ca^2+^ transient peak, (*D*) Ca^2+^ transient amplitude, (*E*) caffeine-induced Ca^2+^ transient amplitude, and (*F*) SERCA activity (RZ, *n* = 76,8 hearts) (BZ *n* = 43,8 hearts). (*G*) Typical Ca^2+^ transients 1-day post-MI in C57BL/6J mice. Mean (*H*) Ca^2+^ transient peak, (*I*) Ca^2+^ transient amplitude, (*J*) caffeine-induced Ca^2+^ transient amplitude, and (*K*) SERCA activity (RZ, *n* = 64,7 hearts), (BZ *n* = 30,7 hearts). (*L*) Typical Ca^2+^ transients 1-day post-MI in *Runx1*^Δ/Δ^-mice. Mean (*M*) Ca^2+^ transient peak, (*N*) Ca^2+^ transient amplitude, (*O*) caffeine-induced Ca^2+^ transient amplitude, and (*P*) SERCA activity (RZ, *n* = 16,4 hearts) (BZ *n* = 9,4 hearts). Error bars = mean ± SEM. **P < 0.01, ***P < 0.001. SEM, standard error of the mean.

SR-mediated calcium release leads to sarcomere shortening and contraction, which are key processes determining LV pump function. Differences in BZ and RZ cardiomyocyte SR-mediated calcium release in the first-week post-MI contribute to regional heterogeneity of contractile function across the LV.^9,14,15^ This regional heterogeneity contributes to impaired global LV contractile function and poor patient prognosis.^8–10^*Runx*1 expression in BZ cardiomyocytes is increased as early as 1-day following MI,^21^ at which time LV contractile function is also substantially reduced.^1–4^ The most severe contractile dysfunction in the surviving myocardium is in the BZ (where *Runx*1 expression is highest) and the least dysfunction is in the RZ (where *Runx*1 expression is lowest).^6,11,12^ Whether this change in *Runx*1 expression simply coincides with, or impacts on, SR-mediated calcium handling was unknown. We have previously shown that genetically modified cardiomyocyte-specific *Runx*1-deficient mice (*Runx*1^Δ/Δ^) have preserved LV cardiac contractility at 1-day post-MI.^21^ Therefore, we compared SR calcium handling in BZ and RZ cardiomyocytes isolated from the hearts of C57BL/6J and *Runx*1^Δ/Δ^ mice (and *Runx*1^fl/fl^ controls)^21^ at 1-day post-MI (*Figure* 1*A*). These results were compared to cardiomyocytes isolated from C57BL/6J hearts without MI at equivalent regions to where the ‘BZ’ and ‘RZ’ would be found post-MI, for which there were no regional differences in calcium handling, as expected (*Figure* 1*B–F*; all raw values are reported in see Supplementary material online, *Table S1*). Cardiomyocytes were stimulated at 1.0 Hz to elicit SR-mediated calcium release into the cytosol (calcium transients), the amplitude of which largely determines the force of contraction (*Figure 1A*).

In BZ cardiomyocytes, the calcium transient peak (systolic [Ca^2+^]_i_) following MI was 64% of the peak in RZ cardiomyocytes (*P* < 0.05; *Figure 1G* and *H*). In *Runx*1^Δ/Δ^ hearts, the calcium transient peak was preserved in BZ cardiomyocytes relative to RZ cardiomyocytes (*P* > 0.05; *Figure 1L* and *M*). There was no difference in the diastolic [Ca^2+^]_i_ (calcium transient minimum) between BZ and RZ in all groups (*P* > 0.05; see Supplementary material online, *Figure S2A–C*). The changes in systolic [Ca^2+^]_i_ in BZ cardiomyocytes following MI resulted in a calcium transient amplitude that was 47% of that observed in RZ cardiomyocytes (*P* < 0.05; *Figure 1G* and *I*). By contrast, the calcium transient amplitude in BZ cardiomyocytes of *Runx*1^Δ/Δ^ hearts was preserved (*P* > 0.05; *Figure 1L* and *N*).

The SR calcium content is the predominant determinant of calcium transient amplitude.^23,24^ To determine the SR calcium content, a rapid bolus of caffeine (10 mmol/L) was applied at the end of the protocol (*Figure 1A*) to release all the calcium from the SR into the cytosol, permitting the quantification of the SR calcium content. In BZ cardiomyocytes at 1-day post-MI, the caffeine-induced Ca^2+^ transient amplitude (SR calcium content) was 71% of that observed in RZ cardiomyocytes (*P* < 0.05; *Figure 1J*). The SR calcium content of *Runx*1^Δ/Δ^ BZ cardiomyocytes was not significantly different from that of RZ cardiomyocytes of the same hearts (*P* > 0.05; *Figure 1O*).

A key determinant of the SR calcium content is uptake into the SR via the calcium ATPase pump SERCA.^25^ We hypothesized that the lowered SR calcium content in BZ cardiomyocytes following MI might reflect decreased SERCA activity (K_SERCA_) or enhanced extrusion of calcium from the cell via the sodium-calcium exchanger. To determine K_SERCA_, we measured the rate constant of decay of the caffeine-induced calcium transient (which includes sarcolemmal efflux but not SR calcium uptake) and subtracted this value from that of the electrically stimulated calcium transient (which includes both SR calcium uptake and sarcolemmal efflux).^26,27^ After MI, K_SERCA_ of BZ cardiomyocytes was 48% of RZ cardiomyocytes (*P* < 0.05; *Figure 1K*). However, SERCA activity was not different between *Runx*1^Δ/Δ^ BZ and RZ cardiomyocytes 1-day following MI (*P* > 0.05; *Figure 1P*). Extrusion of calcium from the cell via the sodium-calcium exchanger was assessed by the time constant of caffeine-induced calcium transient decay but was not different between the BZ and RZ cardiomyocytes of any group (see Supplementary material online, *Table.S1*).

Separate experiments confirmed that the relative difference between BZ and RZ in all parameters measured in C57BL/6J mice at 1-day post-MI (*Figure 1G–K*) were also observed in *Runx*1*^fl/fl^* mice at 1-day post-MI (see Supplementary material online, *Table S1*).

These data demonstrate that marked differences in SR-mediated calcium release/uptake between BZ and RZ cardiomyocytes that are known to contribute to whole heart contractile dysfunction in the first week post-MI, are evident as early as 1-day post-MI in both C57BL/6J and cre-negative littermate controls and are *Runx*1-dependent.

### 3.2 *Runx*1 inhibits expression of genes involved in oxidative phosphorylation

**Figure 2:**
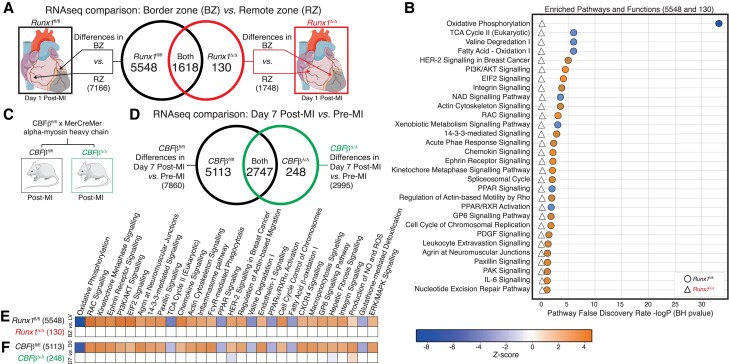
*Runx1* regulates oxidative phosphorylation. (*A*) Comparison and Venn diagram of gene differences in *Runx1*^Δ/Δ^ and *Runx1^fl^*^/fl^-mice (*n* = 6). (*B*) Enriched biological pathways ranked by logP using IPA from unique differences between BZ and RZ in *Runx1^fl^*^/fl^ (circles) compared with BZ and RZ differences in *Runx1*^Δ/Δ^-mice (triangles). The heatmap (blue to orange) represents predicted inhibition and activation or no change of pathways based on the Z-score. (*C*) Generation of *Cbfβ*^Δ/Δ^ and *Cbfβ^fl^*^/fl^-mice. (*D*) Comparison and Venn diagram of gene expression in *Cbfβ*^Δ/Δ^ and *Cbfβ^fl^*^/fl^-mice (*n* = 6). (*E*) The enriched biological pathway from IPA generated from unique differences and (*F*) *Cbfβ*-mice ranked on Z-score.

**Figure 3:**
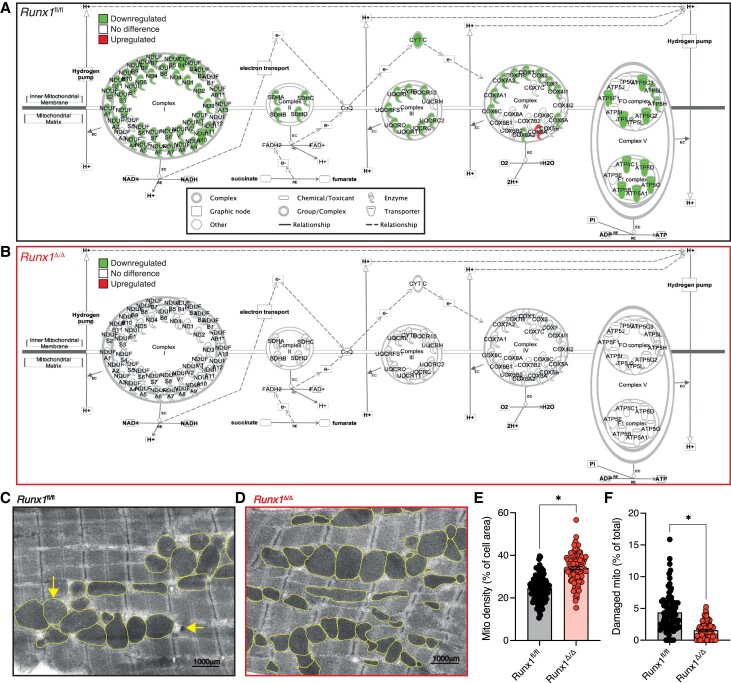
*Runx1* deficiency protects mitochondria post-MI. (*A*) Genes involved in oxidative phosphorylation from BZ and RZ 1-day post-MI in IPA of *Runx1^fl^*^/fl^ (*n* = 6, 7166 differentially expressed genes) and (*B*) *Runx1*^Δ/Δ^-mice (*n* = 6, 1748 differentially expressed genes). (*C*) Electron microscopy images from BZ of *Runx1^fl^*^/fl^ and (*D*) *Runx1*^Δ/Δ^-mice. (*E*) Quantification of mitochondrial density (percentage of cell area) and (*F*) number of damaged mitochondria from the BZ from *Runx1^fl^*^/fl^ and *Runx1*^Δ/Δ^-mice (*n* = 92 cells; two hearts) 1-day post-MI. Error bars = mean ± SEM. *P < 0.05. SEM, standard error of the mean.

The BZ is a discrete and highly active region of the myocardium post-MI that undergoes a considerable number of transcriptional changes, some of which ultimately may drive adverse cardiac remodelling. The extent to which the observed increase of *Runx*1 within the BZ plays a role in these transcriptional changes is unknown. Therefore, we used RNAseq to compare the changes in gene expression in the BZ myocardium relative to the RZ in both *Runx*1^Δ/Δ^ and *Runx*1*^fl/fl^* control mice at 1-day post-MI. Using a false discovery rate (FDR) cut-off of ≤0.05, there were 7166 differentially expressed genes in the BZ relative to the RZ in *Runx*1*^fl/fl^* control mice (*Figures 2A and 3A*). By contrast, there were only 1748 differentially expressed genes in the BZ relative to the RZ in *Runx*1^Δ/Δ^ mice. There were 1618 differentially expressed genes that were common to both *Runx*1^Δ/Δ^ and *Runx*1*^fl/fl^* control mice leaving 5548 differentially expressed genes unique to *Runx*1*^fl/fl^* but only 130 differentially expressed genes unique to *Runx*1^Δ/Δ^ mice (*Figure 2A*, see Supplementary material online, *Figure S3B*). Ingenuity pathway analysis (IPA) software used Z-score and FDR to determine the enriched pathways and functions specific to the 5548 differentially expressed genes unique to *Runx*1*^fl/fl^* mice, as compared to the 130 differentially expressed genes unique to the *Runx*1^Δ/Δ^ mice (*Figure 2B*). Oxidative phosphorylation was the most highly significant changed pathway/function (*Figure 2B*).

To confirm this new discovery linking *Runx*1 to genes regulating oxidative phosphorylation we used an alternative approach to inhibit *Runx*1 in cardiomyocytes by targeting its essential co-factor CBFβ. Reduced *Cbf*β expression in *Cbf*β^Δ/Δ^ mice was confirmed using qPCR (see Supplementary material online, *Figure S3C* and *D*). RUNX1 partners with CBFβ to act as a heterodimeric transcription factor.^18^ Interfering with RUNX1 binding to CBFβ significantly reduces affinity for RUNX1 binding to its target genes thus its transcriptional activity.^28^ Whole LV myocardium taken before and 7-days after MI was compared in both *Cbf*β^Δ/Δ^ and *Cbf*β*^fl/fl^* control mice. Using an FDR of 0.05, there were 7860 differentially expressed genes in *Cbf*β^fl/fl^ control mice and only 2995 differentially expressed genes in *Cbf*β^Δ/Δ^ mice of which 2747 were common to both, leaving 5113 differentially expressed genes unique to *Cbf*β*^fl/fl^* mice and not significantly affected in *Cbf*β^Δ/Δ^ (*Figure 2D*, see Supplementary material online, *Figure S3E* and *F*). These overall numbers are very similar to those shown in *Runx*1^Δ/Δ^ mice (*Figure 2A*). In IPA, Z-score was used to determine and compare the enriched pathways and functions in the gene changes unique to *Runx*1^Δ/Δ^ mice (*Figure 2E*) to the gene changes unique to the *Cbf*β^Δ/Δ^ mice (*Figure 2F*). As with our finding with *Runx*1^Δ/Δ^ mice (*Figure 2B* and *E*), oxidative phosphorylation was the most significantly changed pathway (*Figure 2F*), further confirming the ability of RUNX1 to regulate genes involved in oxidative phosphorylation.

Interrogation of these pathways using IPA analysis revealed marked downregulation of genes involved in oxidative phosphorylation across all five inner mitochondrial complexes in BZ myocardium of *Runx*1^fl/fl^ control mice compared to RZ (*Figure 3A*, see Supplementary material online, *Figure S4*). By contrast, no genes involved in oxidative phosphorylation within mitochondria were significantly downregulated in the BZ of *Runx*1^Δ/Δ^ mice suggesting that *Runx*1 deficiency within cardiomyocytes preserves genes involved in oxidative phosphorylation within the BZ following MI (*Figure 3B* and see Supplementary material online, *Figure S4* left). In *Cbf*β^fl/fl^ mice, 87% of genes involved in oxidative phosphorylation revealed marked downregulation (see Supplementary material online, *Figures S4* and *S5A*). By contrast, only 47% of mitochondrial genes involved in oxidative phosphorylation were downregulated in the *Cbf*β^Δ/Δ^ mice demonstrating that cardiomyocyte deficiency in CBFβ or RUNX1 preserves genes involved in oxidative phosphorylation following MI (see Supplementary material online, *Figures S4* and *S5B*).

Mitochondria are critical for energy production in the heart via ATP generation from the process of oxidative phosphorylation. Given the marked preservation of expression of genes involved in oxidative phosphorylation within the BZ of *Runx*1^Δ/Δ^ mice, we next quantified mitochondrial density, integrity, and size from electron microscopy images taken from the BZ region of *Runx*1^fl/fl^ and *Runx*1^Δ/Δ^ mice at 1-day post-MI. The BZ of *Runx*1^fl/fl^ mice had reduced mitochondrial density compared to the BZ of *Runx*1^Δ/Δ^ mice (24 and 34% of cell area respectively, *P* < 0.05; *Figure 3C–E*). Furthermore, there were significantly more damaged mitochondria (identified by dissolved/damaged cristae or mitophagosomes) in the BZ of *Runx*1^fl/fl^ mice compared to the BZ of *Runx*1^Δ/Δ^ mice (4.4% and 1.5% of all mitochondria respectively, *P* < 0.05; *Figure 3F*, see Supplementary material online, *Figure S6A and B*). No difference in mitochondrial size was detectable between groups (see Supplementary material online, *Figure S6C*).

### 3.3 Targeting *Runx*1 protects cardiac function

**Figure 4:**
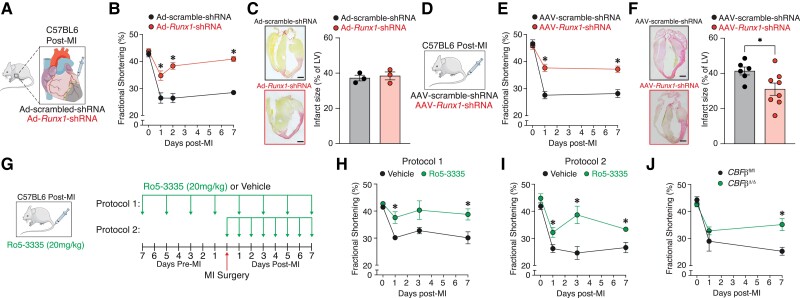
Targeting *Runx1* protects cardiac function post-MI. (*A*) Schematic of Ad. (*B*) Echocardiographic data for FS of Ad-*Runx1*-shRNA vs. Ad-scramble-shRNA. (*C*) Typical picrosirius-red-stained hearts and infarct size as the percentage of the LV. (*D*) Schematic of AAV. (*E*) FS of AAV-*Runx1*-shRNA vs. AAV-scramble-shRNA. (*F*) Typical picrosirius-red stained hearts and infarct size as a percentage of the LV AAV-*Runx1*-shRNA (*n* = 8) vs. AAV-scramble-shRNA (*n* = 6). (*G*) Protocols for Ro5-3335. (*H*) FS for mice receiving protocol 1 and (*I*) protocol 2. (*J*) FS from *Cbf*β^Δ/Δ^-MI vs. *Cbf*β^fl/fl^-MI mice. Error bars = mean ± SEM. *P < 0.05. SEM, standard error of the mean.

Whether increased *Runx*1 expression post-MI in the BZ cardiomyocytes can be therapeutically targeted remains unknown. To address this gap in our knowledge we utilized various approaches. The first was to inject an adenoviral (Ad) vector expressing *Runx*1-shRNA (or control Ad-scrambled-shRNA) directly into the BZ area of C57BL/6J mice immediately following MI (*Figure 4A*) to reduce *Runx*1 expression and determine the impact on LV contractility. Experiments were performed to confirm the ability of the shRNA to knock-down *Runx*1 in BZ cardiomyocytes and are detailed in the methods (see Supplementary material online, *Figures S7, S8A* and *C*). Echocardiography was performed before and after MI to assess LV function (*Figure 4B*, see Supplementary material online, *Figure S8E, Table S2*). As expected, there was a decline in cardiac systolic function (measured by fractional shortening; %FS) post-MI in the Ad-scramble-shRNA injected control group of mice. However, the Ad-*Runx*1-shRNA group had a markedly preserved contractile function at all time points post-MI relative to the Ad-scramble-shRNA injected control group (day 1, 34.7 ± 1.8 vs. 26.4 ± 1.9; day 2, 38.3 ± 1.3 vs. 26.4 ± 1.7; and day 7, 40.9 ± 0.9 vs. 28.5 ± 0.6% FS; *P* < 0.05; *Figure 4B*). To determine whether a change in infarct size contributed to preserved cardiac function, Sirius red staining was performed on heart slices at 7-days post-MI (*Figure 4C*). Infarct size was not different between the Ad-*Runx*1-shRNA and Ad-scramble-shRNA injected control group (*P* > 0.05; *Figure 4C*).

Although knockdown of *Runx*1 in the BZ myocardium was effective in preserving cardiac contractile function, the need to directly inject the heart with the Ad may have reduced the translational potential of the approach. We, therefore, explored the use of a cardiotropic adeno-associated virus serotype 9 (AAV9) expressing a shRNA targeting *Runx*1, which can be injected intravascularly to knockdown *Runx*1 within the heart. AAV vectors are widely used for cardiac gene delivery in pre-clinical models and clinical trials^29^ and a licensed AAV9 gene therapy is available for the treatment of spinal muscular atrophy.^30^ AAV9 encoding shRNA provides highly efficient knockdown in the heart.^31^ RNAscope was performed to confirm the ability of the shRNA to knock-down *Runx*1 in BZ cardiomyocytes and are detailed in the methods (see Supplementary material online, *Figure S8B* and *D*), AAV9-*Runx*1-shRNA or control AAV-scramble-shRNA were delivered via tail-vein injection following MI in C57BL/6J mice (*Figure 4D*). Echocardiography was performed before and after MI (*Figure 4E*). As with direct Ad delivery, the AAV9-*Runx*1-shRNA group had a markedly preserved contractile function post-MI relative to the AAV9-scramble-shRNA injected control group (day-1, 37.7 ± 1.4 vs. 27.6 ± 1.4; day 7; 37.2 ± 1.2 vs. 28.2 ± 1.5% FS; *P* < 0.05, *Figure 4E*). In contrast to direct injection of Ad-*Runx*1-shRNA into the BZ, intravenous injection of AAV9-*Runx*1-shRNA reduced infarct size in C57BL/6J-MI mice relative to AAV9-scramble-shRNA group post-MI at 7 days (41.4 ± 1.9 vs. 31.1 ± 3.5% of LV; *P* < 0.05; *Figure 4F*).

We then utilized an established small molecule-specific inhibitor of RUNX1, the benzodiazepine derivative Ro5-3335, which alters the interaction between RUNX1 and CBFβ (reducing the functional activity of RUNX1^28^) in two different protocols as an alternative therapeutic approach to gene transfer in the context of MI. The first protocol involved pre-treatment with Ro5-335 in C57BL/6J mice every other day for 7-days followed by the same treatment pattern following MI, whereas the second protocol involved post-treatment with Ro5-3335 in C57BL/6J mice daily for 7-days following MI (*Figure 4G*). *Runx*1 expression in LV myocardium was decreased relative to vehicle control, consistent with studies that have used Ro5-3335 to inhibit *Runx*1 in other tissues^32^ (*P* < 0.05; see Supplementary material online, *Figure S8G*). Following MI, echocardiography showed the expected sustained decline in cardiac systolic function (measured by %FS) in the vehicle-injected groups (*Figure 4H* and *I*). By contrast, in the Ro5-3335 injected groups, protocol 1 mice had a preserved contractile function at days 1 and 7-days post-MI relative to the vehicle control group (day-1, 37.6 ± 2.2 vs. 30.1 ± 0.6; day 3, 40.4 ± 3.5 vs. 32.8 ± 1.2; day 7; 38.8 ± 2.0 vs. 30.1 ± 2.2% FS; *P* < 0.05, *Figure 4H*) and protocol 2 mice had a preserved contractile function at all time points post-MI relative to the vehicle control group (day 1, 32.2 ± 1.8 vs. 26.2 ± 1.5; day 3, 38.6 ± 3.2 vs. 24.6 ± 2.4; day 7; 33.3 ± 0.6 vs. 26.6 ± 1.9% FS; *P* < 0.05, *Figure 4I*).

We next sought to confirm our results with Ro5-3335 using our cardiac-specific CBFβ-deficient (*Cbf*β^Δ/Δ^) mice and littermate controls (*Cbf*β^fl/fl^), which provided an organ-specific approach to limit the potential for CBFβ to interact with RUNX1 (*Figure 2C*). Echocardiography, performed before and after MI, demonstrated a sustained decline in cardiac systolic function (measured by %FS) in *Cbf*β^fl/fl^ mice (*Figure 4J*). By contrast, *Cbf*β^Δ/Δ^ mice demonstrated a preserved contractile function at day 7 post-MI relative to control *Cbf*β^fl/fl^ mice (35.2 ± 2.2 vs. 25.3 ± 1.5% FS; *P* < 0.05, *Figure 4J*). These data support the findings with Ro5-3335 that limiting RUNX1 function by reducing the availability of CBFβ in cardiomyocytes preserves cardiac function.

In separate experiments, we also confirmed that Ro5-3335 was effective in the context of ischemia-reperfusion using an *ex vivo* Langendorff-perfused rat heart preparation (see Supplementary material online, *Figure S9A*). Ro5-3335 (1 µM) improved LV-developed pressure post-reperfusion to 186 ± 6.3% relative to vehicle control (see Supplementary material online, *Figure 9B* and *C*).

These data confirm that both gene therapeutic and pharmacological approaches to inhibit RUNX1 preserve cardiac contractile function following MI.

## 4. Discussion

In this study, we demonstrate the significant role of RUNX1 in directing early pathological changes in the BZ region post-MI, including an impact on the energy-generating machinery of cardiomyocytes. Additionally, we show that *Runx*1 is targetable by gene therapy or a small molecule drug, indicating potential for clinical translation, including RUNX1 inhibitors as a new class of drug to prevent cardiac contractile dysfunction.

Further, we show that abnormal calcium handling in BZ cardiomyocytes occurs as early as 1-day post-MI, which is driven by the increase in *Runx*1 in the BZ. The abnormalities include a reduced calcium transient amplitude, SR calcium content, and sarco/endoplasmic reticulum Ca²⁺-ATPase (SERCA) activity in BZ cardiomyocytes relative to cardiomyocytes isolated from the RZ region. These data are supported by previous studies that demonstrate that the BZ region has reduced SERCA expression, calcium transient amplitude, and contraction at 3–7 days following MI.^13,33^ In contrast, the amplitude of calcium release, SR calcium content, and SERCA activity was not significantly different between the BZ and RZ region in *Runx*1-deficient mice post-MI demonstrating homogeneous calcium handling across regions that is likely to contribute to the improved LV contractile function observed in *Runx*1-deficient mice at 1-day following MI. Our previous results at the later time point of 2 weeks post-MI demonstrated a role for *Runx*1 in determining SERCA-mediated calcium uptake, SR calcium content, and calcium transient amplitude.^21^ The current study provides new insight in that this function of RUNX1 is evident in BZ cardiomyocytes as early as 1-day post-MI.

Myocardial infarction is commonly characterized by changes in energy/substrate metabolism and decreased mitochondrial function with abnormalities of ATP production being more severe in the BZ relative to the RZ.^17,34^ This pattern, which occurs across species (rodents, sheep, pigs, zebrafish, and humans), coincides with a parallel reduction in the expression of genes associated with oxidative phosphorylation, reduced BZ contractile function, and progression to HF.^17,35–39^ Our study provides new insight demonstrating that RUNX1 in the BZ drives a reduction in the expression of genes associated with mitochondrial oxidative phosphorylation as early as 1-day post-MI, potentially contributing to impaired BZ contractile function. Our electron microscopy revealed that 1-day following MI, mitochondrial density is reduced and architecture is impaired in the BZ. By contrast, the BZ of our *Runx*1-deficient mice had preserved expression of genes associated with mitochondrial oxidative phosphorylation relative to the RZ and displayed both greater mitochondrial density and structural integrity relative to control hearts post-MI. Mitochondrial size (and, therefore, potentially changes to mitochondrial fusion/fission) was not altered by *Runx*1.

Given the potential of *Runx*1-deficiency in cardiomyocyte-specific genetically modified mice to improve two key indices of BZ cardiomyocyte contractility (*i.e.* calcium handling and mitochondrial function), we tested whether targeting RUNX1 expression/activity in the BZ of wild-type mice could enhance contractility post-MI. We first used two viral vector-mediated gene delivery approaches of *Runx*1-shRNA to knockdown *Runx*1 within the BZ. Both approaches resulted in a marked preservation of LV contractile function post-MI. The level of improved contractility was equivalent to that observed in cardiomyocyte-specific *Runx*1-deficient mice.^21^ One unexpected difference between the two approaches was that, in contrast to direct BZ myocardial injection of Ad-*Runx*1-shRNA, intravenous injection of AAV9-*Runx*1-shRNA produced a reduction in infarct size following MI. It is unclear why this difference in infarct size was only present when using AAV9-*Runx*1-shRNA but it is unlikely to explain the preservation of contractile function since direct myocardial BZ injection of Ad-*Runx*1-shRNA produced the same level of preserved LV contractility without a change in infarct size.

Cardiac contractile function was preserved post-MI when Ro5-3335 was delivered either before or immediately after MI (the latter being a potential translational approach). Ro5-3335 has previously been shown to improve pulmonary hypertension and retinal angiogenesis by altering vascular remodelling, endothelial to haemopoietic transition, and pulmonary macrophage activity.^40,41^ Given the ability of Ro5-3335 to inhibit RUNX1-dependent processes outside the heart it was important to determine that the preservation in contractile function observed in Ro5-3335 treated mice was related to a direct effect on cardiomyocytes. Therefore, we firstly used Ro5-3335 to improve cardiac contractile function in isolated hearts and secondly developed a tamoxifen-inducible cardiomyocyte-specific *Cbf*β-deficient mouse to limit *Runx*1 activity in cardiomyocytes in a similar way to Ro5-3335. *Cbf*β-deficient mice also demonstrated preserved LV contractile function thus confirming (alongside our four other approaches to inhibit RUNX1 within the heart) that inhibiting RUNX1 within cardiomyocytes is a therapeutically tractable approach with translational potential to preserve cardiac contractile function following MI. Similar to our findings in *Runx*1-deficient mice, the *Cbf*β-deficient mice had preserved expression levels of oxidative phosphorylation genes post-MI, thus highlighting a common RUNX1-dependent mechanism observed in more than one of our approaches to inhibit RUNX1 activity in cardiomyocytes.

Although this study focuses on RUNX1 in the context of MI our findings have broader implications for RUNX1 biology in other tissues and diseases (cardiac and non-cardiac).^19^ Increased RUNX1 expression is now observed in a wide range of cardiac^20,42–45^ and non-cardiac diseases (e.g. retinal vascular dysfunction,^41^ liver disease,^46^ septic shock,^47^ and kidney dysfunction^48^) and link RUNX1 to processes (e.g. oxidative stress, apoptosis, and fibrosis) that contribute to cardiac remodelling.^49–51^ It is becoming increasingly clear that RUNX1 inhibitors may represent a future therapy for a wide range of diseases including MI.^52,53^

## 5. Clinical perspectives and translational outlook

### 5.1 Competency in medical knowledge

The architecture and function of the heart change over time in people who survive a MI. This progressive transformation leads to deterioration of cardiac contractility and HF; an outcome affecting 26 million people worldwide for which there is no cure.

### 5.2 Translational outlook 1

We can intervene by targeting RUNX1 in the BZ and stop this process from happening thus addressing one of the biggest challenges in cardiac research.

### 5.3 Translational outlook 2

Increased *Runx*1 expression has been observed in a wide range of cardiac and non-cardiac diseases. Thus, it has become increasingly clear that RUNX1 inhibitors could offer a future therapy for a variety of diseases. Our findings forge the way for translational research using RUNX1 inhibitors.

### 5.4 Translational outlook 3

Given the importance of our novel data linking increased *Runx*1 expression to mitochondrial number and genes involved in oxidative phosphorylation, this study opens up the potential to explore the importance of RUNX1 in cellular processes/diseases where mitochondrial oxidative phosphorylation gene expression is demonstrated to be decreased; for example, mitochondrial disorders and age-associated organ dysfunction.

## 6. Future research & limitations

The new link between RUNX1 and mitochondrial function has profound implications for cardiac contractility and patient outcome and warrants future investigation. For example, the extent to which the expression of genes associated with oxidative phosphorylation in the BZ of *Runx*1-deficient mice post-MI is regulated by effects of RUNX1 on mitochondria number (via an altered balance of mitochondrial destruction/biogenesis), a multitude of genes, or a key regulator remains an intriguing area to explore.

There are some technical limitations to be considered in this work. Although M-mode echocardiography is a well-established method for the evaluation of contractile function, it is restricted, and two-dimensional measurements would provide a more robust functional analysis. Furthermore, we did not directly interrogate the off-target effects of Ro5-3335, which may be an important direction for future studies.

Despite this convincing evidence that RUNX1 contributes to the pathophysiology of MI *via* effects on oxidative phosphorylation and Ca^2+^-handling, it is possible that additional mechanisms may contribute, such as an effect of RUNX1 on autophagy, apoptosis, or inflammation which has previously been demonstrated in other contexts.

Given that RUNX1 is a master transcription factor that controls multiple cellular processes, we anticipate the potential for non-cardiomyocyte effects of small molecule-based RUNX1 inhibition (e.g. vascular remodelling) that may contribute also to improved LV function. Indeed, using RUNX1 as a multi-targeted approach to cardioprotection could be more beneficial than single-target-based therapy to prevent adverse cardiac remodelling.^54^

## Supplementary material

Supplementary material is available at *Cardiovascular Research* online.

## Supplementary Material

cvad107_Supplementary_Data

## Data Availability

The data underlying this article will be shared on reasonable request to the corresponding author.
