# Supplementary material for: Ribonucleicacid interference or small molecule inhibition of Runx1 in the border zone prevents cardiac contractile dysfunction following myocardial infarction
Source: Cardiovasc Res. 2023 Jul 11;119(16):2663–71. doi: 10.1093/cvr/cvad107 (PMC10730241; doi:10.1093/cvr/cvad107)
Supplement: cvad107_Supplementary_Data [file cvad107_supplementary_data.zip › EXTENDED_FIGS.pdf]

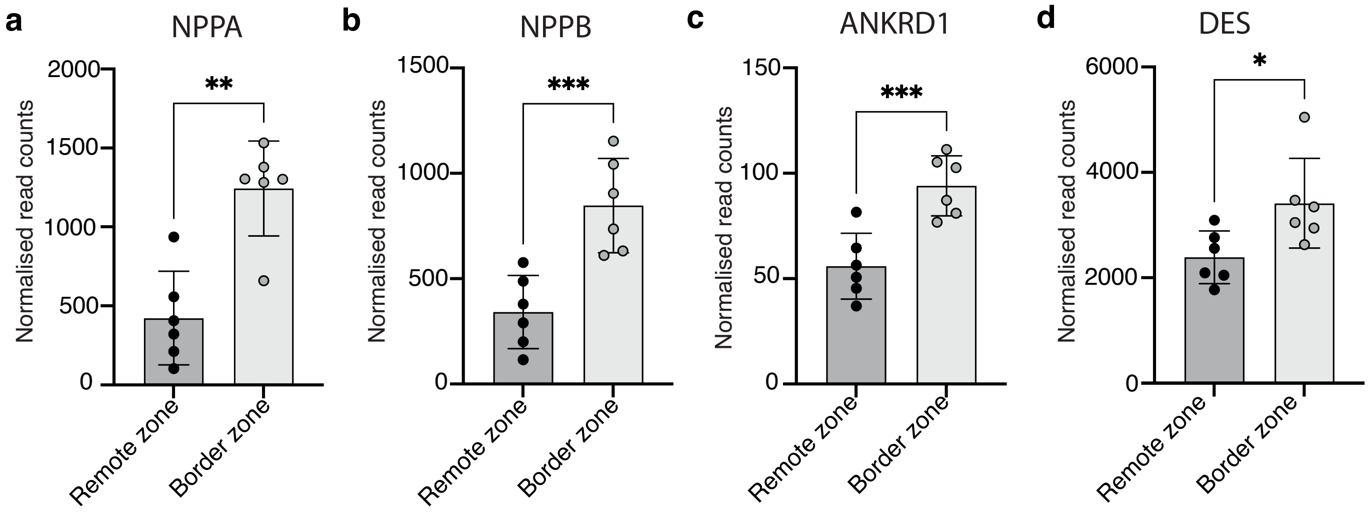


**Extended Figure 1. Confirmation that markers for the border zone are enriched within the dissected border zone region.** RNAseq data confirmed the enrichment of (a) NPPA, (b) NPPB, (c) ANKRD1 and (d) DES in the meticulously isolated border zone region relative to the remote left ventricular zone (remote zone), confirming the specificity and consistency of our approach for isolating border zone tissue.

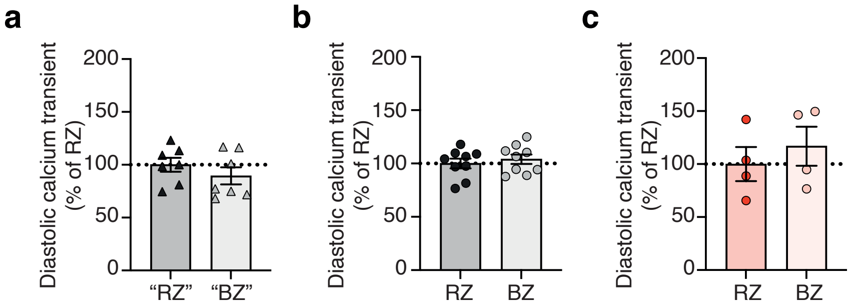


**Extended Figure 2. Diastolic calcium transient in *Runx1*$^{\Delta/\Delta}$ mice and C57BL/6J mice 1 day post myocardial infarction (MI).** **(a)** Diastolic calcium ($Ca^{2+}$) transient before MI in C57BL6 mice in the remote zone (RZ) *n*=76 (7 hearts) and border zone (BZ) *n*=43 (hearts). **(b)** Diastolic $Ca^{2+}$ transient at 1 day post-MI in C57BL6 mice in the RZ *n*=64 (9 hearts) and BZ *n*=30 (9 hearts). **(c)** Diastolic $Ca^{2+}$ transient at 1 day post-MI in *Runx1*$^{\Delta/\Delta}$ mice in the RZ *n*=16 (4 hearts) and BZ *n*=9 (4 hearts). Error bars represent mean ± SEM, $P>0.05$.

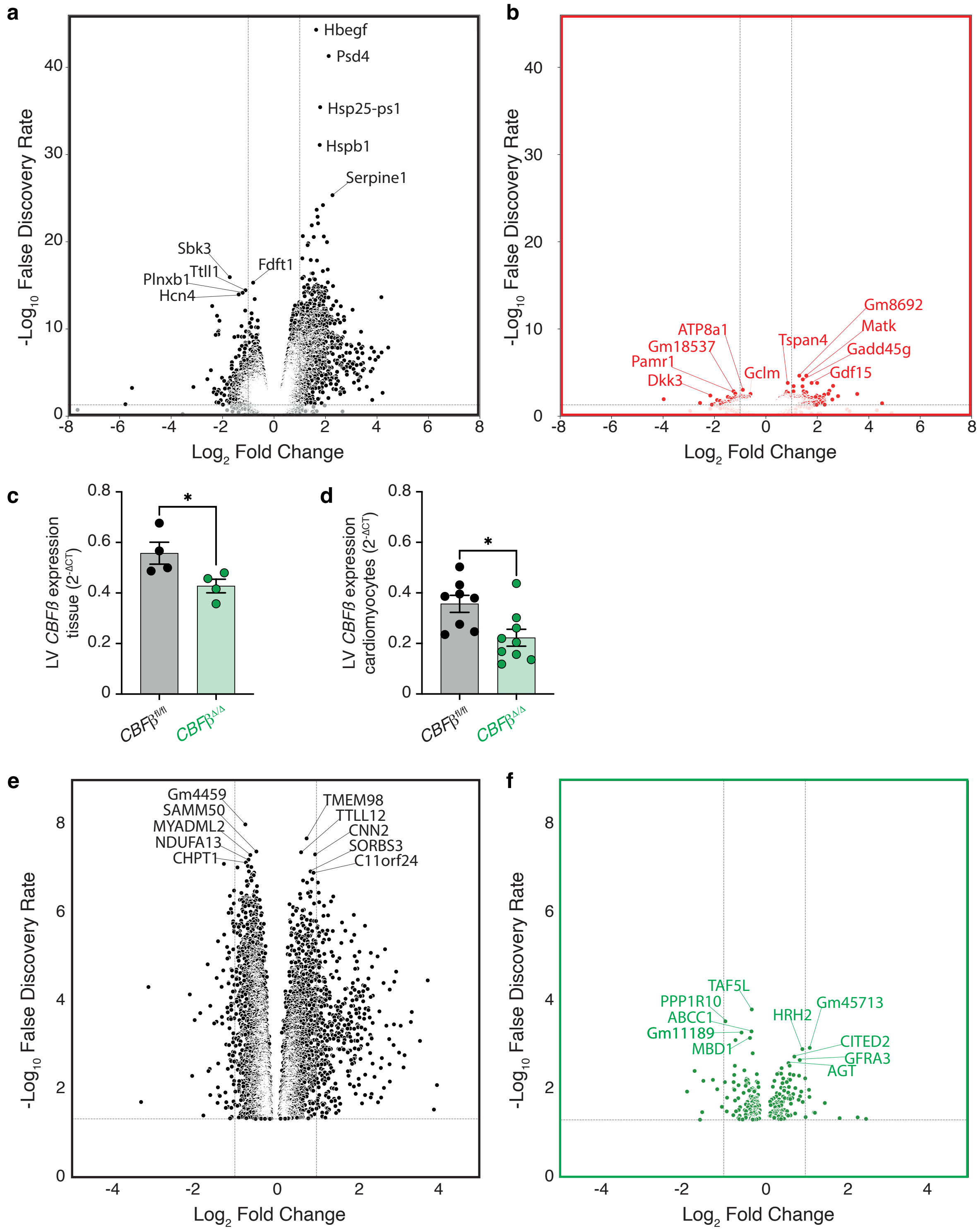


**Extended Figure 3. Volcano plots in the border zone (BZ) and remote zone (RZ) in *Runx1*$^{fl/fl}$, *Runx1*$^{\Delta/\Delta}$ mice 1 day post-myocardial infarction (MI) and *Cbfβ* expression and volcano plots in *Cbfβ*$^{fl/fl}$ and *Cbfβ*$^{\Delta/\Delta}$ mice 7 days post-MI.** Volcano plots of differentially regulated genes unique to **(a)** *Runx1*$^{fl/fl}$ mice (left, black, *n*=6) and **(b)** *Runx1*$^{\Delta/\Delta}$ mice (right, red, *n*=6) in the BZ versus the RZ at 1 day post-MI. Top five upregulated and down regulated genes based on false discovery rate (FDR) are noted. **(c)** *Cbfβ* mRNA expression relative to *Ppib* as measured by qPCR in whole LV tissue from *Cbfβ* $^{\Delta/\Delta}$ MI mice (*n*=4) vs. *Cbfβ*$^{fl/fl}$ MI mice (*n*=4). **(d)** *Cbfβ* mRNA expression relative to *Ppib* as measured by qPCR in LV isolated cardiomyocytes from *Cbfβ*$^{\Delta/\Delta}$ MI mice (*n*=9) vs. *Cbfβ* $^{fl/fl}$ MI mice (*n*=8). *P<0.05, Student *t* test. Volcano plots of differentially regulated genes in whole LV tissue unique to **(e)** *Cbfβ*$^{fl/fl}$ mice (left, black, *n*=6) and **(f)** *Cbfβ*$^{\Delta/\Delta}$ mice (right, green, *n*=6) 7 days post-MI compared to pre-MI. Top five upregulated and down regulated genes based on false discovery rate (FDR) noted.

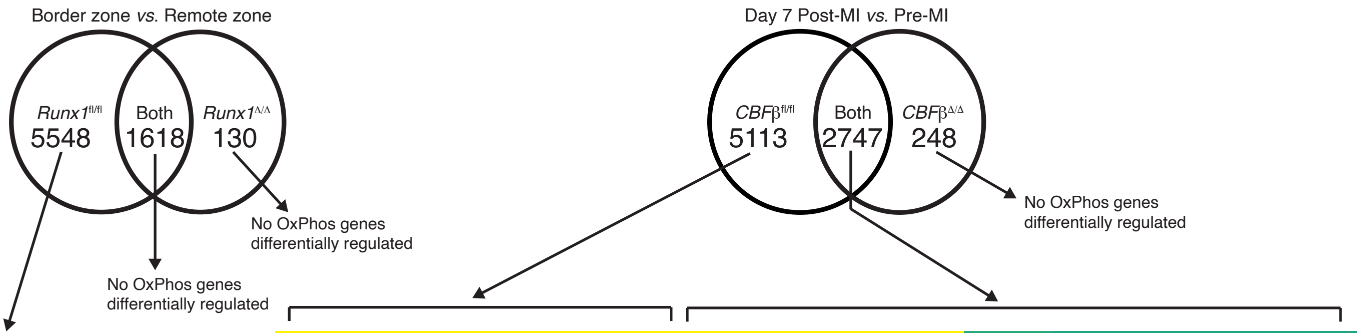


### *Runx1*$^{fl/fl}$

| | FDR | Log Ratio | Intensity |
|---|---|---|---|
| ATP5A1 | 0.0039523 | -0.76 | 11.5 |
| ATP5B | 0.0022955 | -0.67 | 11.9 |
| ATP5C1 | 0.0116387 | -0.63 | 9.5 |
| ATP5D | 0.0058572 | -0.47 | 8.6 |
| ATP5F1 | 0.0092230 | -0.63 | 9.0 |
| ATP5G2 | 0.0480540 | -0.25 | 6.6 |
| ATP5G3 | 0.0029577 | -0.72 | 9.5 |
| ATP5H | 0.0359252 | -0.46 | 9.1 |
| ATP5J2 | 0.0474604 | -0.40 | 8.6 |
| ATP5L | 0.0105228 | -0.52 | 7.6 |
| ATP5O | 0.0073731 | -0.45 | 8.9 |
| COX1 | 0.0016757 | -0.80 | 16.4 |
| COX2 | 0.0010174 | -0.65 | 13.9 |
| COX3 | 0.0010280 | -0.75 | 14.2 |
| COX4I1 | 0.0004623 | -0.62 | 9.3 |
| COX5A | 0.0020023 | -0.64 | 9.1 |
| COX6A1 | 0.0038411 | 0.33 | 5.8 |
| COX6A2 | 0.0129188 | -0.52 | 9.9 |
| COX6C | 0.0235638 | -0.47 | 5.4 |
| COX7A1 | 0.0191882 | -0.60 | 8.6 |
| COX7A2 | 0.0229588 | -0.41 | 7.6 |
| COX7B | 0.0305723 | -0.47 | 8.9 |
| CYTB | 0.0122457 | -0.63 | 15.3 |
| ND1 | 0.0003733 | -0.83 | 14.4 |
| ND2 | 0.0022125 | -0.73 | 13.9 |
| ND3 | 0.0017862 | -0.57 | 10.1 |
| ND4 | 0.0022339 | -0.71 | 14.4 |
| ND4L | 0.0031282 | -0.87 | 11.1 |
| ND5 | 0.0052253 | -0.80 | 14.3 |
| NDUFA1 | 0.0231656 | -0.43 | 7.0 |
| NDUFA10 | 0.0032834 | -0.69 | 8.5 |
| NDUFA11 | 0.0466337 | -0.37 | 5.9 |
| NDUFA13 | 0.0229270 | -0.39 | 8.2 |
| NDUFA2 | 0.0381739 | -0.29 | 7.1 |
| NDUFA4 | 0.0062586 | -0.47 | 8.5 |
| NDUFA5 | 0.0127572 | -0.63 | 7.8 |
| NDUFA6 | 0.0378071 | -0.44 | 6.6 |
| NDUFA7 | 0.0418919 | -0.27 | 6.9 |
| NDUFA8 | 0.0016706 | -0.66 | 7.6 |
| NDUFA9 | 0.0110475 | -0.59 | 8.6 |
| NDUFAB1 | 0.0045633 | -0.71 | 3.6 |
| NDUFB10 | 0.0243309 | -0.65 | 7.7 |
| NDUFB11 | 0.0338063 | -0.44 | 7.8 |
| NDUFB2 | 0.0443747 | -0.39 | 6.7 |
| NDUFB3 | 0.0319472 | -0.42 | 6.6 |
| NDUFB4 | 0.0214261 | -0.47 | 4.1 |
| NDUFB5 | 0.0196200 | -0.53 | 7.4 |
| NDUFB6 | 0.0082544 | -0.48 | 6.6 |
| NDUFB7 | 0.0174701 | -0.36 | 7.5 |
| NDUFB8 | 0.0382447 | -0.41 | 7.9 |
| NDUFB9 | 0.0063922 | -0.55 | 8.6 |
| NDUFS1 | 0.0090476 | -0.75 | 9.2 |
| NDUFS2 | 0.0041991 | -0.65 | 9.4 |
| NDUFS3 | 0.0070739 | -0.66 | 7.7 |
| NDUFS4 | 0.0103826 | -0.58 | 6.8 |
| NDUFS6 | 0.0208048 | -0.46 | 7.5 |
| NDUFS7 | 0.0002880 | -0.74 | 7.8 |
| NDUFS8 | 0.0159283 | -0.40 | 7.2 |
| NDUFV1 | 0.0013644 | -0.68 | 8.5 |
| NDUFV2 | 0.0130075 | -0.53 | 8.3 |
| NDUFV3 | 0.0413884 | -0.39 | 7.5 |
| SDHA | 0.0034819 | -0.77 | 10.2 |
| SDHB | 0.0107764 | -0.57 | 9.2 |
| SDHC | 0.0005796 | -0.71 | 8.1 |
| SDHD | 0.0120490 | -0.66 | 8.4 |
| UQCR10 | 0.0327368 | -0.41 | 7.7 |
| UQCR11 | 0.0151261 | -0.40 | 7.4 |
| UQCRB | 0.0318067 | -0.46 | 8.2 |
| UQCRC1 | 0.0011091 | -0.72 | 9.4 |
| UQCRC2 | 0.0157486 | -0.61 | 9.4 |
| UQCRFS1 | 0.0055431 | -0.67 | 9.2 |
| UQCRQ | 0.0277681 | -0.36 | 8.1 |

### *CBFβ*$^{fl/fl}$

| | FDR | Log Ratio | Intensity |
|---|---|---|---|
| ATP5D | 0.0000073 | -0.43 | 8.7 |
| ATP5G1 | 0.0000022 | -0.59 | 9.3 |
| ATP5G2 | 0.0000072 | -0.45 | 6.8 |
| ATP5G3 | 0.0000122 | -0.59 | 9.9 |
| ATP5J | 0.0000055 | -0.77 | 8.6 |
| ATP5J2 | 0.0000322 | -0.65 | 8.9 |
| ATP5L | 0.0121140 | -0.72 | 7.8 |
| COX1 | 0.0002330 | -0.75 | 16.4 |
| COX2 | 0.0007620 | -0.70 | 13.9 |
| COX3 | 0.0024200 | -0.77 | 14.0 |
| COX4I1 | 0.0018550 | -0.44 | 9.5 |
| COX6A2 | 0.0000268 | -0.61 | 10.0 |
| COX7A2 | 0.0000002 | -0.61 | 8.2 |
| CYTB | 0.0001400 | -0.75 | 15.1 |
| ND1 | 0.0028610 | -0.75 | 14.2 |
| ND2 | 0.0003630 | -0.80 | 13.7 |
| ND3 | 0.0200290 | -0.63 | 10.1 |
| ND4 | 0.0004380 | -0.77 | 14.3 |
| ND4L | 0.0005770 | -0.90 | 10.7 |
| ND5 | 0.0003310 | -0.83 | 14.0 |
| NDUFA11 | 0.0338320 | -0.51 | 0.0 |
| NDUFA12 | 0.0000033 | -0.68 | 7.6 |
| NDUFA13 | 0.0000001 | -0.66 | 8.4 |
| NDUFA2 | 0.0000064 | -0.54 | 7.4 |
| NDUFA4 | 0.0000604 | -0.69 | 8.9 |
| NDUFA5 | 0.0000006 | -0.72 | 8.0 |
| NDUFA6 | 0.0000006 | -0.61 | 6.8 |
| NDUFA7 | 0.0000249 | -0.58 | 7.0 |
| NDUFA8 | 0.0000004 | -0.53 | 8.1 |
| NDUFAB1 | 0.0000059 | -0.69 | 3.7 |
| NDUFB10 | 0.0010650 | -0.68 | 8.1 |
| NDUFB6 | 0.0000001 | -0.55 | 6.9 |
| NDUFS4 | 0.0091320 | -0.44 | 7.2 |
| NDUFS7 | 0.0000022 | -0.56 | 8.1 |
| NDUFV3 | 0.0000485 | -0.60 | 7.6 |
| UQCR11 | 0.0000039 | -0.68 | 7.6 |

| *CBFβ*$^{fl/fl}$ FDR | Log Ratio | Intensity | | *CBFβ*$^{\Delta/\Delta}$ FDR | Log Ratio | Intensity |
|---|---|---|---|---|---|---|
| 0.00000009 | -0.73 | 11.7 | ATP5A1 | 0.0146323 | -0.29 | 11.7 |
| 0.00000003 | -0.69 | 12.0 | ATP5B | 0.0054477 | -0.29 | 12.0 |
| 0.00000005 | -0.71 | 9.7 | ATP5C1 | 0.0306031 | -0.23 | 9.7 |
| 0.00000028 | -0.70 | 9.3 | ATP5F1 | 0.0437999 | -0.24 | 9.3 |
| 0.00000043 | -0.59 | 9.4 | ATP5H | 0.0183580 | -0.24 | 9.4 |
| 0.00000491 | -0.62 | 9.1 | ATP5O | 0.0321916 | -0.28 | 9.1 |
| 0.00000046 | -0.60 | 9.4 | COX5A | 0.0360319 | -0.22 | 9.4 |
| 0.00000127 | -0.53 | 8.7 | COX6B1 | 0.0035017 | -0.30 | 8.7 |
| 0.00000068 | -0.71 | 5.5 | COX6C | 0.0215390 | -0.30 | 5.5 |
| 0.00000002 | -1.11 | 8.9 | COX7A1 | 0.0017462 | -0.52 | 8.9 |
| 0.00000013 | -0.69 | 9.1 | COX7B | 0.0411036 | -0.23 | 9.1 |
| 0.01793716 | -0.12 | 6.7 | COX8A | 0.0492167 | -0.11 | 6.7 |
| 0.00000002 | -0.72 | 7.1 | NDUFA1 | 0.0039720 | -0.31 | 7.1 |
| 0.00000036 | -0.60 | 8.6 | NDUFA10 | 0.0072908 | -0.28 | 8.6 |
| 0.00000184 | -0.68 | 7.3 | NDUFA3 | 0.0353796 | -0.28 | 7.3 |
| 0.00000002 | -0.73 | 8.8 | NDUFA9 | 0.0016777 | -0.35 | 8.8 |
| 0.00002809 | -0.66 | 7.2 | NDUFB1 | 0.0161446 | -0.38 | 7.2 |
| 0.00000002 | -0.67 | 8.2 | NDUFB11 | 0.0033537 | -0.29 | 8.2 |
| 0.00000399 | -0.61 | 7.0 | NDUFB2 | 0.0387330 | -0.26 | 7.0 |
| 0.00000344 | -0.50 | 7.0 | NDUFB3 | 0.0112614 | -0.26 | 7.0 |
| 0.00000002 | -0.74 | 4.3 | NDUFB4 | 0.0205777 | -0.26 | 6.9 |
| 0.00000002 | -0.64 | 7.7 | NDUFB5 | 0.0085608 | -0.25 | 7.7 |
| 0.00000041 | -0.52 | 7.8 | NDUFB7 | 0.0408524 | -0.19 | 7.8 |
| 0.00000031 | -0.63 | 8.3 | NDUFB8 | 0.0061780 | -0.30 | 8.3 |
| 0.00000005 | -0.67 | 8.9 | NDUFB9 | 0.0421062 | -0.21 | 8.9 |
| 0.00000001 | -0.83 | 9.5 | NDUFS1 | 0.0008842 | -0.41 | 9.5 |
| 0.00000004 | -0.67 | 9.6 | NDUFS2 | 0.0078584 | -0.27 | 9.6 |
| 0.00000065 | -0.68 | 8.0 | NDUFS3 | 0.0150749 | -0.30 | 8.0 |
| 0.00000020 | -0.70 | 7.9 | NDUFS6 | 0.0221499 | -0.27 | 7.9 |
| 0.00000014 | -0.65 | 7.5 | NDUFS8 | 0.0121548 | -0.27 | 7.5 |
| 0.00000064 | -0.65 | 8.6 | NDUFV1 | 0.0322420 | -0.25 | 8.6 |
| 0.00000074 | -0.66 | 8.7 | NDUFV2 | 0.0382739 | -0.25 | 8.7 |
| 0.00000018 | -0.87 | 10.4 | SDHA | 0.0149728 | -0.36 | 10.4 |
| 0.00000010 | -0.69 | 9.4 | SDHB | 0.0162599 | -0.27 | 9.4 |
| 0.00000019 | -0.67 | 8.4 | SDHC | 0.0200184 | -0.26 | 8.4 |
| 0.00000007 | -0.64 | 8.8 | SDHD | 0.0144046 | -0.25 | 8.8 |
| 0.00000073 | -0.45 | 8.0 | UQCR10 | 0.0171973 | -0.20 | 8.0 |
| 0.00000003 | -0.69 | 8.5 | UQCRB | 0.0079927 | -0.27 | 8.5 |
| 0.00000008 | -0.66 | 9.7 | UQCRC1 | 0.0461082 | -0.21 | 9.7 |
| 0.00000004 | -0.65 | 9.6 | UQCRC2 | 0.0029839 | -0.30 | 9.6 |
| 0.00000014 | -0.72 | 9.5 | UQCRFS1 | 0.0089345 | -0.31 | 9.5 |
| 0.00005959 | -0.58 | 2.1 | UQCRH | 0.0106783 | -0.38 | 2.1 |
| 0.00000048 | -0.66 | 8.4 | UQCRQ | 0.0277046 | -0.26 | 8.4 |

Downregulated

Upregulated

**Extended Figure 4.** Genes involved in oxidative phosphorylation from RNAseq Ingenuity pathway analysis (IPA). List of genes downregulated (green) or upregulated (red) in oxidative phosphorylation complexes from RNAseq IPA pathway analysis for each group (*n*=6 for each). There were no oxidative phosphorylation (OxPhos) genes altered in the border zone vs. remote zone of *Runx1*$^{\Delta/\Delta}$ mice, so all changes were unique to *Runx1*$^{fl/fl}$. Between Day 7 post-MI and pre-MI there were changes in *CBFβ*$^{\Delta/\Delta}$ mice (turquoise box), however all changes were shared with *CBFβ*$^{fl/fl}$. There were also genes downregulated in *CBFβ*$^{fl/fl}$ mice but not *CBFβ*$^{\Delta/\Delta}$ mice (i.e. unique changes), all genes changed in *CBFβ*$^{fl/fl}$ mice (shared and unique) are contained within the yellow box.

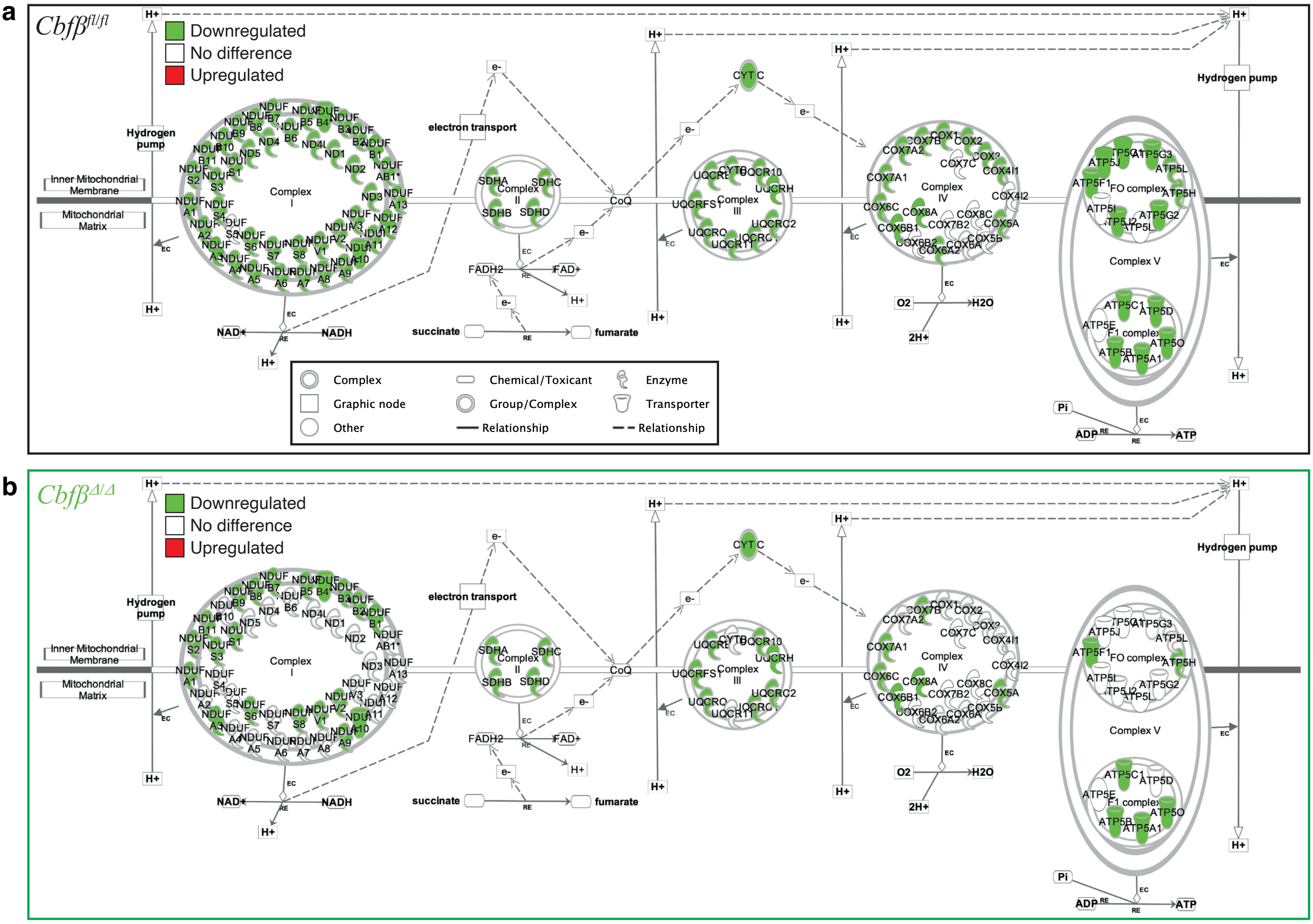


**Extended Figure 5. Oxidative phosphorylation genes in the border zone (BZ) of *Cbfβ*$^{fl/fl}$ and *Cbfβ*$^{\Delta/\Delta}$ mice 7 days post-myocardial infarction (MI).** Schematic visualisation of genes involved in oxidative phosphorylation complexes from the RNAseq data from the whole LV of *Cbfβ*$^{fl/fl}$ (*n*=6) and *Cbfβ*$^{\Delta/\Delta}$ (*n*=6) mice 7 days post-MI in Ingenuity Pathway Analysis (IPA) (downregulated – green, upregulated – red, no difference – white) of **(a)** *Cbfβ*$^{fl/fl}$ mice (7860 differentially expression genes) and **(b)** *Cbfβ*$^{\Delta/\Delta}$ mice (2995 differentially expressed genes).

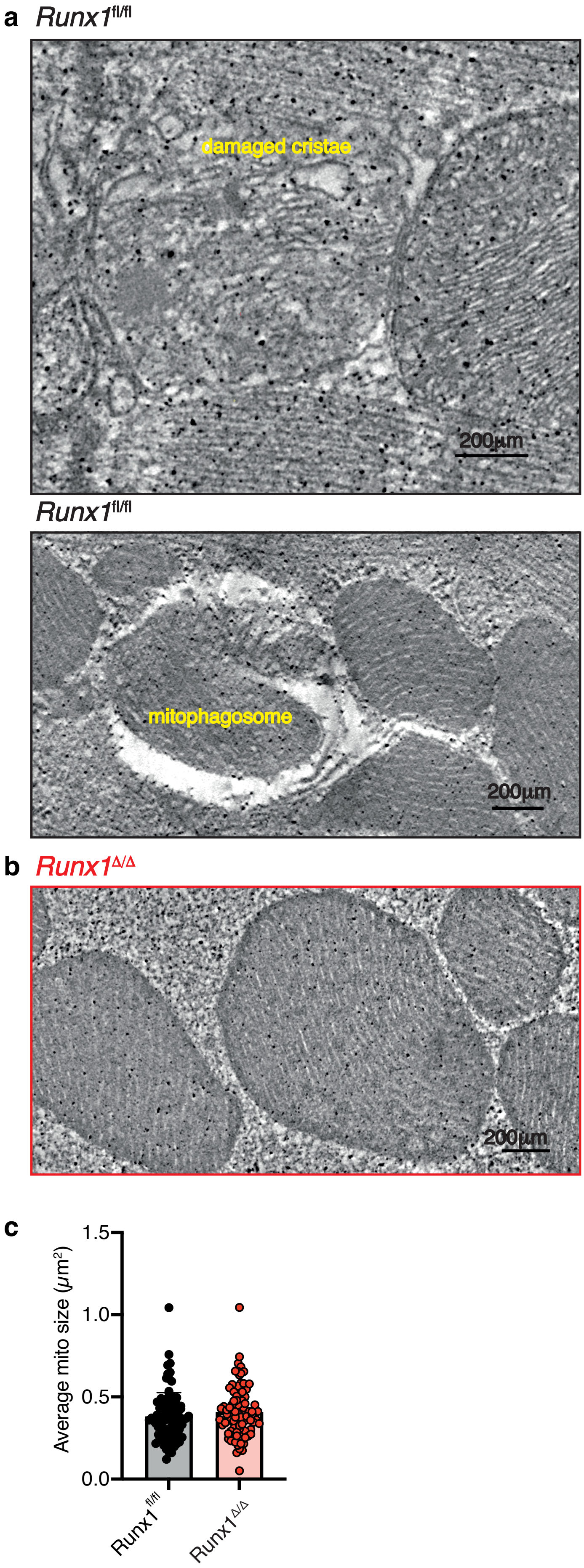


**Extended Figure 6. Mitochondrial size in the border zone (BZ) of *Runx1$^{fl/fl}$* and *Runx1$^{\Delta/\Delta}$* mice 1 day post-myocardial infarction (MI).** High-resolution electron tomography representative images of **(a)** damaged mitochondria from the BZ of *Runx1$^{fl/fl}$* mice 1 day post-MI and **(b)** healthy mitochondria from the BZ of *Runx1$^{\Delta/\Delta}$* mice 1 day post-MI. **(c)** Quantification of the average mitochondrial size from electron microscopy images of the BZ from *Runx1$^{fl/fl}$* mice (*n*=92 cells from 2 hearts) and *Runx1$^{\Delta/\Delta}$* mice (*n*=92 cells from 2 hearts) 1 day post-MI. Error bars represent mean ± SEM. *$P$<0.05, unpaired Student *t*-test on average heart data.

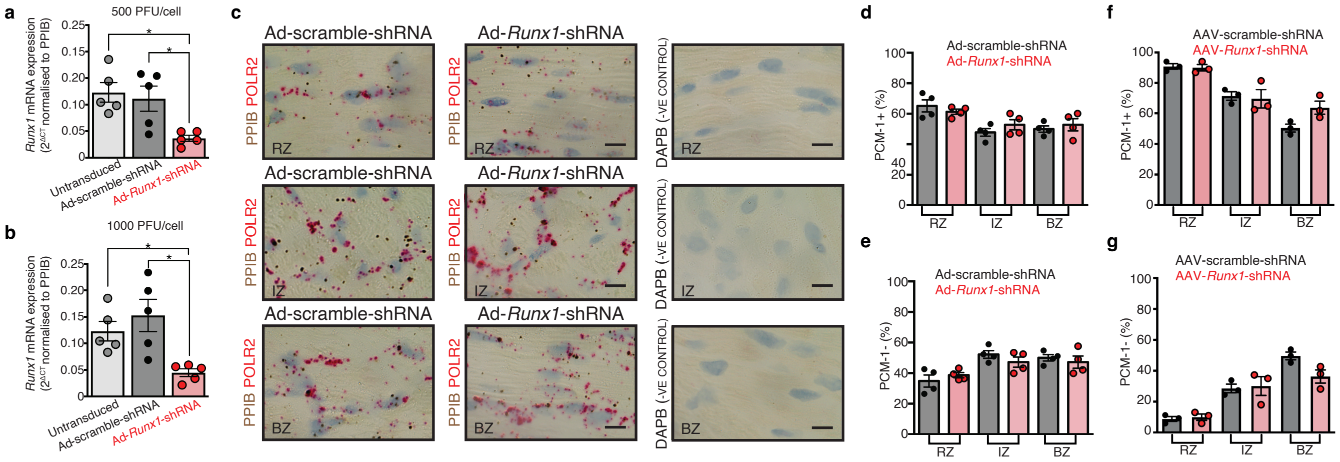


**Extended Figure 7. *Runx1* mRNA expression in IP1B cells transduced with Ad-*Runx1*/scramble- shRNA and control RNAscope images and cardiomyocyte and non-cardiomyocyte expression using Pericentriolar material 1 (PCM-1).** *Runx1* mRNA expression relative to *Peptidylprolyl Isomerase B (Ppib)* measured by real-time quantitative polymerase chain reaction in IP1B cells transduced with Ad- *Runx1*-shRNA (*n*=5; biological replicates) vs. Ad-scramble-shRNA (*n*=5; biological replicates) at **(a)** 500 PFU/cell or **(b)** 1000 PFU/cell. Error bars represent mean ± SEM. **P*<0.05, Student *t* test. RNA in situ hybridisation using RNAscope at 7 days post-myocardial infarction (MI) in Ad-*Runx1*-shRNA (*n*=4) vs. Ad-scramble-shRNA (*n*=4) and at 7 days post-MI in AAV-*Runx1*-shRNA (*n*=3) vs. AAV-scramble-shRNA (*n*=3). **(c)** Positive control slides using *Ppib* (red puncate dots) and Polr2 (brown punctate dots) probes in both Ad-*Runx1*/scramble-shRNA MI mice and negative controls using bacterial bacillus subtilis dihydodipicolinate reductase (dapB). **(d)** Mean quantification of PCM-1 positive (cardiomyocyte) nuclei expressed as the percentage of total nuclei in Ad- *Runx1*/scramble-shRNA mice and **(e)** mean quantification of PCM-1 negative (non-cardiomyocyte) nuclei expressed as a percentage of total nuclei Ad-Runx1/scramble-shRNA mice. **(f)** Mean quantification of PCM-1 positive (cardiomyocyte) nuclei expressed as the percentage of total nuclei in AAV- Runx-1/scramble-shRNA mice. **(g)** Mean quantification of PCM-1 negative (non-cardiomyocyte) nuclei expressed as the percentage of total nuclei AAV-Runx1/scramble-shRNA mice. Error bars represent mean ±SEM.

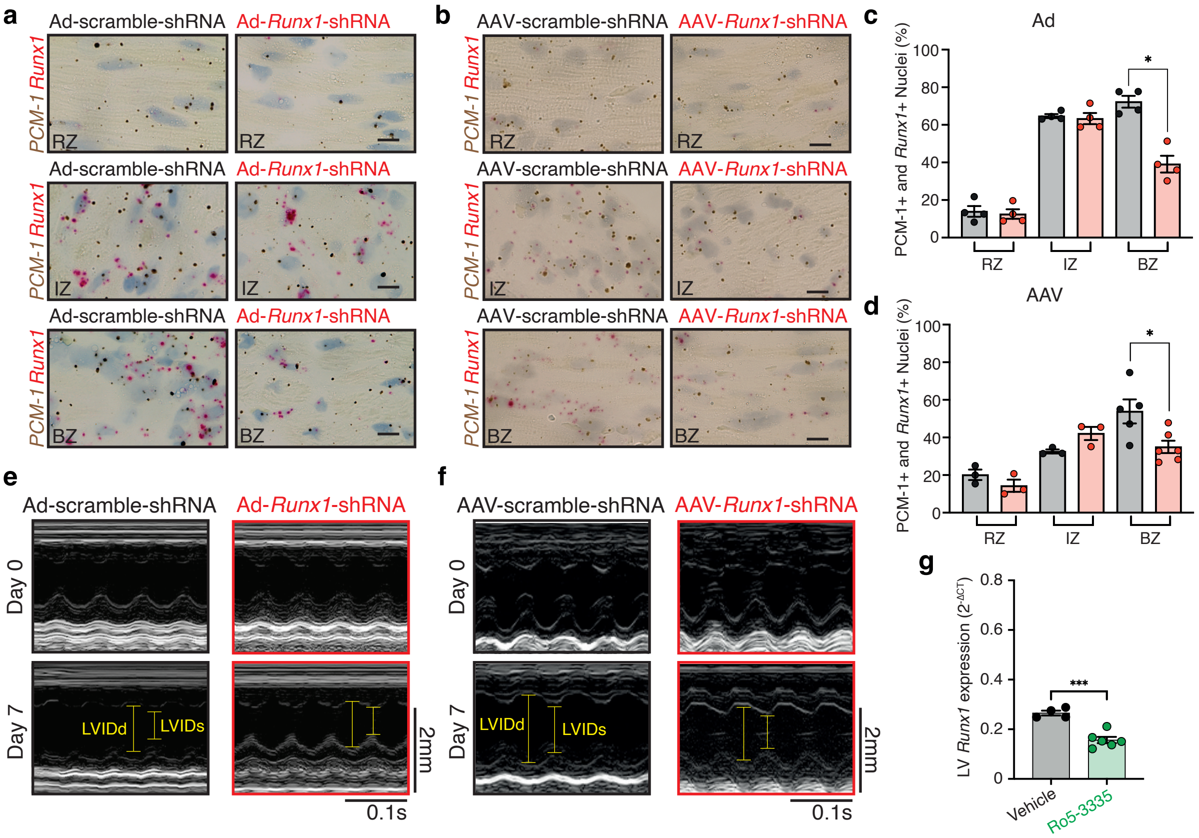


**Extended Figure 8. Runx1 expression in Ad/AAV-*Runx1*-shRNA and Ro5-3335 injected mice. (a-b)** Typical images of regional heart sections by RNA in situ hybridisation (using RNAscope). Regions examined were the remote zone (RZ), infarct zone (IZ) and border zone (BZ) at 7 days post-MI in Ad-*Runx1*-shRNA injected mice (*n*=4) and Ad-scramble-shRNA injected mice (*n*=4) **(a)** and in the RZ (AAV-*Runx1*-shRNA [*n*=3] vs. AAV-scramble-shRNA injected mice [*n*=3]), IZ (AAV-*Runx1*-shRNA [*n*=3] vs. AAV-scramble-shRNA injected mice [*n*=3]), and BZ (AAV-*Runx1*-shRNA [*n*=6] vs. AAV-scramble-shRNA injected mice [*n*=5]) **(b)**, at 7 days post-MI. Probes for *Runx1* (red punctate dots) and pericentriolar material 1 (PCM-1) (brown punctate dots) were used. Scale bar, 10 µm; magnification 100x. **(c)** Mean quantification of *Runx1*-positive cardiomyocyte nuclei (PCM-1+ and *Runx1*) expressed as a percentage of total nuclei 7 days after MI (Ad-*Runx1*-shRNA vs. Ad-scramble-shRNA, **P*<0.05). **(d)** Mean quantification of *Runx1* positive cardiomyocyte nuclei (PCM- 1+ and *Runx1*) expressed as a percentage of total nuclei 7 days after MI (AAV-*Runx1*-shRNA vs. AAV-scramble-shRNA, **P*<0.05). **(e-f)** Echocardiography (scale: x=0.1 s; y=2 mm). **(g)** *Runx1* mRNA expression relative to *Peptidylprolyl Isomerase B (Ppib)* as measured by real-time quantitative polymerase chain reaction (qPCR) in LV tissue from Ro5-3335-treated MI mice (*n*=6) vs. vehicle (DMSO)-treated MI mice (*n*=4). ****P*<0.005, Student t test.

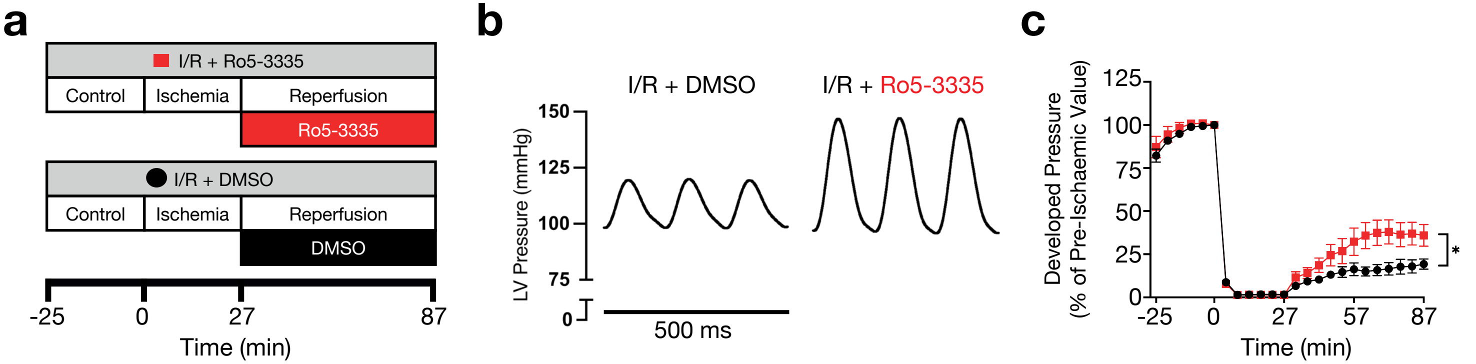


**Extended Figure 9.** Cardiac function in ex vivo rat hearts during IR injury treated upon reperfusion with the Ro5-3335. (A) Schematic of the protocol used. (B) Typical LV pressure measured in heart treated with either IR+ DMSO or IR+ Ro5-3335 (C) Developed LV pressure data for IR+DMSO (n = 8) and IR+Ro5-3335 (n =8 ). *$P < 0.05$. Data are expressed as mean ± standard error of the mean (SEM). Statistical comparisons were made by a two-sample Student's *t*-test on the raw data.

**Extended Table 1 - Calcium**

| | pre-MI C57BL6 | | | | Day 1 Post-MI C57BL6 | | | | Day 1 Post-MI Runx1 deficient | | | | Day 1 Post-MI Runx1 floxed | | | |
|---|---|---|---|---|---|---|---|---|---|---|---|---|---|---|---|---|
| | RZ | | BZ | | RZ | | BZ | | RZ | | BZ | | RZ | | BZ | |
| | **MEAN** | *SEM* | **MEAN** | SEM | **MEAN** | *SEM* | **MEAN** | *SEM* | **MEAN** | *SEM* | **MEAN** | SEM | **MEAN** | *SEM* | **MEAN** | *SEM* |
| CALCIUM TRANSIENT PEAK (nmol.L$^{-1}$) | **405.53** | *19.35* | **406.01** | *18.51* | **418.44** | *21.41* | **263.90*** | *19.30* | **317.28** | *17.61* | **373.15** | *67.08* | **540.79** | *40.07* | **313.85*** | *27.34* |
| CALCIUM TRANSIENT MINIMUM (nmol.L$^{-1}$) | **166.46** | *7.50* | **171.96** | *8.04* | **166.38** | *10.92* | **144.35** | *6.66* | **131.96** | *21.18* | **164.08** | *46.72* | **218.32** | *18.17* | **173.90** | *10.10* |
| CALCIUM TRANSIENT AMPLITUDE (nmol.L$^{-1}$) | **239.07** | *16.12* | **234.05** | *16.82* | **252.06** | *19.47* | **119.54*** | *16.92* | **185.26** | *6.24* | **209.07** | *27.10* | **322.47** | *24.51* | **139.95*** | *19.56* |
| CAFFEINE-INDUCED CALCIUM TRANSIENT AMPLITUDE (nmol.L$^{-1}$) | **652.40** | *14.51* | **663.28** | *30.62* | **606.65** | *27.49* | **431.51*** | *43.18* | **457.62** | *82.25* | **474.42** | *79.50* | **413.29** | *18.66* | **315.88*** | *28.84* |
| SERCA ACTIVITY (KSERCA (s$^{-1}$)) | **10.35** | *0.77* | **9.11** | *0.96* | **9.62** | *1.21* | **4.51*** | *0.76* | **6.16** | *0.68* | **7.53** | *2.02* | **6.56** | *0.57* | **4.63*** | *0.40* |
| TIME CONSTANT OF CAFFEINE INDUCED TRANSIENT DECAY (NCX ACTIVITY (s$^{-1}$)) | **1.16** | *0.10* | **0.98** | *0.08* | **1.01** | *0.21* | **1.24** | *0.39* | **0.72** | *0.14* | **0.72** | *0.13* | **0.41** | *0.02* | **0.41** | *0.02* |
| CALCIUM TRANSIENT PEAK TO MINIMUM RATIO | **2.45** | *0.12* | **2.38** | *0.11* | **2.56** | *0.16* | **1.84*** | *0.12* | **2.53** | *0.26* | **2.57** | *0.40* | **2.49** | *0.08* | **1.80*** | *0.09* |

**Extended Table 1.** Raw data quantifying calcium ($Ca^{2+}$) handling parameters. $Ca^{2+}$ transient peak (nmol.L$^{-1}$), $Ca^{2+}$ transient minimum (nmol.L $^{-1}$), $Ca^{2+}$ transient amplitude (nmol.L $^{-1}$), caffeine-induced $Ca^{2+}$ transient amplitude (nmol.L $^{-1}$), SERCA activity (KSERCA, s $^{-1}$ ), time constant of caffeine-induced transient decay (NCX activity, s $^{-1}$ ) and calcium transient peak to minimum ratio in C56BL/6J mice before MI in the remote zone (RZ; *n*=76 cardiomyocytes [9 hearts]) and border zone (BZ; *n*=43 cardiomyocytes, [9 hearts]) and after MI in the RZ (*n*=64 cardiomyocytes, [7 hearts]) and BZ (*n*=30 cardiomyocytes, [7 hearts]) and at 1-day post-MI in the RZ (*n*=16 cardiomyocytes, [4 hearts]) and BZ (*n*=9 cardiomyocytes, [4 hearts]) of *Runx1*$^{\Delta/\Delta}$ mice and in the RZ (*n*=20 cardiomyocytes, [5 hearts]) and BZ (*n*=31 cardiomyocytes, [5 hearts]) of *Runx1*$^{fl/fl}$ mice. **P*<0.05 between RZ and BZ. Student *t* test.

**Extended Table 2 - Echocardiography**

| | Ad-scramble-shRNA | | | | | Ad-Runx1-shRNA | | | | |
|---|---|---|---|---|---|---|---|---|---|---|
| | Fractional shortening (%) | LVIDd (mm) | LVIDs (mm) | LVPWd (mm) | LVPWs (mm) | Fractional shortening (%) | LVIDd (mm) | LVIDs (mm) | LVPWd (mm) | LVPWs (mm) |
| Pre-MI | 42.74 | 3.80 | 2.18 | 1.24 | 1.53 | 43.81 | 3.81 | 2.14 | 1.27 | 1.58 |
| 1-day post-MI | 26.43 | 4.12 | 3.03 | 1.14 | 1.27 | 34.73* | 3.68* | 2.41* | 1.20 | 1.38 |
| 2 days post-MI | 26.38 | 4.26 | 3.13 | 1.06 | 1.32 | 38.32* | 3.71* | 2.29* | 1.11 | 1.38 |
| 7 days post-MI | 28.46 | 3.92 | 2.81 | 1.38 | 1.54 | 40.88* | 3.95* | 2.33 | 1.22 | 1.54 |

| | AAV-scramble-shRNA | | | | | AAV-Runx1-shRNA | | | | |
|---|---|---|---|---|---|---|---|---|---|---|
| | Fractional shortening (%) | LVIDd (mm) | LVIDs (mm) | LVPWd (mm) | LVPWs (mm) | Fractional shortening (%) | LVIDd (mm) | LVIDs (mm) | LVPWd (mm) | LVPWs (mm) |
| Pre-MI | 46.58 | 3.55 | 1.91 | 1.31 | 1.80 | 45.92 | 3.61 | 1.96 | 1.19 | 1.65 |
| 1-day post-MI | 27.62 | 4.02 | 2.93 | 1.04 | 1.28 | 37.66* | 3.89 | 2.43* | 1.20 | 1.54 |
| 7 days post-MI | 28.21 | 4.30 | 3.10 | 1.26 | 1.51 | 37.21* | 3.92 | 2.48* | 1.41 | 1.71 |

| | PROTOCOL 1 | | | | | | | | | |
|---|---|---|---|---|---|---|---|---|---|---|
| | Vehicle | | | | | Ro5-3335 | | | | |
| | Fractional shortening (%) | LVIDd (mm) | LVIDs (mm) | LVPWd (mm) | LVPWs (mm) | Fractional shortening (%) | LVIDd (mm) | LVIDs (mm) | LVPWd (mm) | LVPWs (mm) |
| Pre-MI | 41.45 | 3.46 | 2.03 | 1.36 | 1.59 | 42.72 | 3.44 | 1.97 | 1.29 | 1.72 |
| 1-day post-MI | 30.14 | 3.83 | 2.68 | 1.25 | 1.48 | 37.60* | 3.66 | 2.31 | 1.15 | 1.53 |
| 3 days post-MI | 32.75 | 3.35 | 2.26 | 1.50 | 1.85 | 40.35 | 3.71 | 2.26 | 1.42 | 1.77 |
| 7 days post-MI | 30.13 | 3.56 | 2.49 | 1.33 | 1.52 | 38.77* | 3.73 | 2.29 | 1.36 | 1.61 |

| | PROTOCOL 2 | | | | | | | | | |
|---|---|---|---|---|---|---|---|---|---|---|
| | Vehicle | | | | | Ro5-3335 | | | | |
| | Fractional shortening (%) | LVIDd (mm) | LVIDs (mm) | LVPWd (mm) | LVPWs (mm) | Fractional shortening (%) | LVIDd (mm) | LVIDs (mm) | LVPWd (mm) | LVPWs (mm) |
| Pre-MI | 41.83 | 3.29 | 1.92 | 1.33 | 1.67 | 44.80 | 3.32 | 1.83 | 1.25 | 1.64 |
| 1-day post-MI | 26.23 | 3.82 | 2.83 | 1.21 | 1.35 | 32.20* | 3.73 | 2.54 | 1.10 | 1.46 |
| 3 days post-MI | 24.58 | 3.91 | 2.96 | 0.94 | 1.24 | 38.64* | 3.49 | 2.17 | 1.28 | 1.78 |
| 7 days post-MI | 26.60 | 4.09 | 3.01 | 0.93 | 1.15 | 33.35* | 3.77 | 2.52 | 1.24 | 1.74* |

| | *CBF*$\beta^{fl/fl}$ | | | | | *CBF*$\beta^{\Delta/\Delta}$ | | | | |
|---|---|---|---|---|---|---|---|---|---|---|
| | Fractional shortening (%) | LVIDd (mm) | LVIDs (mm) | LVPWd (mm) | LVPWs (mm) | Fractional shortening (%) | LVIDd (mm) | LVIDs (mm) | LVPWd (mm) | LVPWs (mm) |
| Pre-MI | 44.27 | 3.58 | 2.00 | 1.45 | 1.77 | 42.56 | 3.56 | 2.00 | 1.34 | 1.79 |
| 1-day post-MI | 29.05 | 4.02 | 2.87 | 1.22 | 1.53 | 32.85 | 4.21 | 2.85 | 1.23 | 1.58 |
| 7 days post-MI | 25.32 | 4.10 | 3.07 | 1.18 | 1.38 | 35.16* | 3.94 | 2.57 | 1.33 | 1.64 |

**Extended Table 2. Raw Echocardiography parameters.** Mean fractional shortening (FS%), left ventricular diameter at end diastole (LIVDd, mm), left ventricular diameter at end systole (LVIDs, mm), left ventricular posterior wall thickness at end diastole (LVPWd, mm) and left ventricular posterior wall thickness at end systole (LVPWs, mm) in Ad-*Runx1*-shRNA myocardial infarction (MI) mice (*n*=8; day 0, *n*=8; day 1, *n*=7; day 2, *n*=7; day 7) vs. Ad-scramble-shRNA MI mice (*n*=8; day 0, *n*=8; day 1, *n*=7; day 2, *n*=7; day 7). FS, LVIDd, LVIDs, LVPWd and LVPWs in AAV-*Runx1*-shRNA MI mice (*n*=9; day 0, *n*=9; day 1, *n*=9; day 7) vs. AAV-scramble-shRNA MI mice (*n*=10; day 0, *n*=10; day 1, *n*=9; day 7). FS, LVIDd, LVIDs, LVPWd and LVPWs in Ro5-3335 MI mice (Protocol 1: *n*=6; day 0, *n*=6; day 1, *n*=5; day 3, *n*=5; day 7. Protocol 2: *n*=8; day 0, *n*=8; day 1, *n*=6; day 3, *n*=6; day 7) vs. vehicle (DMSO) MI mice (Protocol 1: *n*=6; day 0, *n*=5; day 1, *n*=3; day 3, *n*=4; day 7. Protocol 2: *n*=7; day 0, *n*=8; day 1, *n*=3; day 3, *n*=7; day 7). FS, LVIDd, LVIDs, LVPWd and LVPWs in $Cbf\beta^{\Delta/\Delta}$ MI mice (*n*=8; day 0, *n*=6; day 1, *n*=6; day 7) vs. $Cbf\beta^{fl/fl}$ MI mice (*n*=6; day 0, *n*=;5 day 1, *n*=6; day 7). *$P$<0.05, Student *t* test between Ad-scramble-shRNA *vs*. Ad-*Runx1*-shRNA, AAV-scramble-shRNA *vs*. AAV-*Runx1*-shRNA, vehicle *vs*. Ro5-3335 and $Cbf\beta^{\Delta/\Delta}$ *vs*. $Cbf\beta^{\Delta/\Delta}$.
