# Supplementary material for: Ribonucleicacid interference or small molecule inhibition of Runx1 in the border zone prevents cardiac contractile dysfunction following myocardial infarction
Source: Cardiovasc Res. 2023 Jul 11;119(16):2663–71. doi: 10.1093/cvr/cvad107 (PMC10730241; doi:10.1093/cvr/cvad107)
Supplement: cvad107_Supplementary_Data [file cvad107_supplementary_data.zip › SUPPLEMENTARY FILE_v3.docx]

**RNA interference or small molecule inhibition of Runx1 in the border zone prevents cardiac contractile dysfunction following myocardial infarction**

Tamara P Martin, PhD^1^†, Eilidh A MacDonald, PhD^1^†, Ashley Bradley, PhD^1^†, Holly Watson, MRes^1^, Priyanka Saxena, PhD^1^, Eva A. Rog-Zielinska^2^, Anmar Raheem, Mres^1^, Simon Fisher, MRes^1^, Ali Ali Mohamed Elbassioni^1,3^, Ohood Almuzaini^1^, Catriona Booth, BSc^1^, Morna Campbell, BSc^1^, Alex Riddell, PhD^1^, Pawel Herzyk^4,5^, Karen Blyth, PhD^6,7^, Colin Nixon, MSc^7^, Lorena Zentilin, PhD^8^, Colin Berry, MD, PhD^1^,Thomas Braun, MD, PhD^9^, Mauro Giacca, MD, PhD^8,10^, Martin W McBride, PhD^1^, Stuart A Nicklin, PhD^1^, Ewan R Cameron, BVMS, PhD^11^, Christopher M Loughrey, BVMS, PhD^1^

†These authors contributed equally to this work and as joint first authors can change the order of authorship for the purposes of curriculum vitae.

Affiliations:

^1^ British Heart Foundation Glasgow Cardiovascular Research Centre, School of Cardiovascular & Metabolic Health, University of Glasgow; Glasgow, UK.

^2^ Institute for Experimental Cardiovascular Medicine, University Heart Centre Freiburg/Bad Krozingen, Faculty of Medicine; Freiburg, Germany.

^3^ Department of Cardiothoracic Surgery, Suez Canal University; Egypt.

^4^ Institute of Molecular, Cell and Systems Biology, University of Glasgow; Glasgow, UK.

^5^ Glasgow Polyomics, University of Glasgow, Garscube Campus; Glasgow, UK.

^6^ School of Cancer Sciences, University of Glasgow; Glasgow, UK.

^7^ Cancer Research UK Beatson Institute, Garscube Estate; Glasgow, UK.

^8^ Molecular Medicine Laboratory, International Centre for Genetic Engineering and Biotechnology; Trieste, Italy.

^9^ Department of Cardiac Development and Remodelling, Max Planck Institute for Heart and Lung Research; Bad Nauheim, Germany.

^10^ School of Cardiovascular Medicine and Sciences, King’s College London British Heart Foundation Centre; London, UK.

^11^ School of Biodiversity, One Health and Veterinary Medicine, University of Glasgow; Glasgow, UK.

Address for correspondence,

Christopher Loughrey

School of Cardiovascular and Metabolic Health,

College of Medical, Veterinary and Life Sciences,

Glasgow Cardiovascular Research Centre, University of Glasgow, University Place

Glasgow, G12 8TA

Telephone: 0044 141 330 2753

Email: [Christopher.Loughrey@glasgow.ac.uk](mailto:Christopher.Loughrey@glasgow.ac.uk)

**METHODS**

**Animals**

The care and use of animals were in accordance with the UK Government Animals (Scientific Procedures) Act 1986. All animal procedures were approved by the University of Glasgow Animal Welfare and Ethical Review Body and licensed by the Home Office, UK (project licence no. P05FEIF82). Mice were housed on a 12/12 h light/ dark cycle and fed and watered ad libitum. All animals used were male mice aged 10-12 weeks of age (weight, 25-30 g) and were randomly assigned to experimental groups. Animals were killed by schedule one procedures (cervical dislocation).

**Generation of cardiomyocyte-specific Runx1 and Cbfβ deficient mice**

Runx1^Δ/Δ^ mice were generated as previously described(1). Cbfβ^fl/fl^ mice(2) (The Jackson Laboratory, ME, USA) were crossed with mice expressing tamoxifen-inducible Cre recombinase (MerCreMer) under the control of the cardiac-specific αMHC (α-myosin heavy chain)(3)) to generate the relevant test (Cbfβ^Δ/Δ^) and littermate (Cbfβ^fl/fl^) control groups. Expression of Cbfβ was reduced to 77% and 47% in LV myocardium and isolated cardiomyocytes respectively in Cbfβ^Δ/Δ^ mice relative to control Cbfβ^fl/fl^ mice (Extended Fig. 3c-d).  All mice used in this study were genotyped using rtPCR by *Transnetyx* for presence of the Cre and floxed alleles.

**Echocardiography**

Mice were anaesthetised in a pre-filled induction chamber with 4% isoflurane in 1.0 L/min O_2_ and then transferred to a face mask and maintained at 0.5-1% isoflurane in 1.0 L/min O_2_. Echocardiography was performed using a Siemens ACUSON Seqoula C512 and a 15L8 Transducer.

**Coronary artery ligation**

Thoracotomy and left anterior descending coronary artery permanent ligation were performed on C57BL/6J (Envigo), Runx1-deficient, Cbfβ-deficient and respective floxed control mice aged 10-12 weeks (weight 25–30 g). Mice were initially anaesthetised by 4% isoflourane (Isoflo, Abbott Laboratories, USA) receiving oxygen at 1 L/min.  Mice received preoperative analgesia of 5 mg/kg carprofen (Rimadyl; Pfizer Animal Health, UK) and 0.1 mg/kg buprenorphine (Vetergesic; Reckitt Benkiser Healthcare Ltd, UK) delivered subcutaneously in a single injection made up to 0.4 mL with saline. Mice were endotracheally intubated and mechanically ventilated at 125 breath/min with a tidal volume of 120 μL. Isofluorane was reduced to 3% initially and then gradually reduced throughout the procedure. A 1 cm skin incision was made perpendicular to the sternum and across the rib cage. The skin and thoracic muscles were carefully retracted back, and the intercostal muscles blunt dissected. The pericardial sac was opened and removed to expose the heart and provide access for ligation. The left anterior descending (LAD) coronary artery was ligated with 9-0 non-adsorbable nylon (W2829 Ethilon; Johnson & Johnson, UK) 1.5 mm distal to the left atrial appendage, with accurate ligation confirmed by visualisation of myocardial blanching below the suture. Three 6-0 non-absorbable prolene pre-placed sutures (W8711; Johnson & Johnson, UK) were inserted around the ribs and the lungs re-inflated. The thoracic muscles were returned to original positions and the skin closed with interrupted stitches using 6-0 absorbable vicryl (W9575; Johnson & Johnson, UK).

**Regional Tissue identification**

Regional sections were carefully dissected as follows: the atria, right ventricle and septum were first cut and removed from each heart. The IZ was identified by the clear difference in colour (white to pale pink) and the location of the tied ligature which acted as a confirmatory marker and was cut and removed from each heart. The next 1 mm thick rim of tissue was defined as the BZ and confirmed as such using specific border zone markers(4) (Extended Fig. 1). The next 2 mm thick rim of tissue was discarded and not used for any analysis, and the remaining left ventricular tissue was defined as the remote zone (RZ). The same process was followed for control hearts using an approximation of the standard location of the IZ.

**Calcium measurements**

Cardiomyocytes from C57BL/6J and Runx1-deficient mice were isolated as previously described with some minor differences(1).  Specifically, following perfusion of the heart, regional sections were identified and removed and each region was placed in separate containers and gently mix to release the isolated cardiomyocytes thus ensuring no cross contamination between regions. BZ and remote RZ were loaded with a calcium-sensitive fluorophore (5.0 μmol/L Fura-4F AM, Invitrogen), and perfused during field stimulation (1.0 Hz, 2.0 ms duration, stimulation voltage set to 1.5 times the threshold). The Fura-4F fluorescence ratio (340/380 nm excitation) was measured with a spinning wheel spectrophotometer (Cairn Research Ltd; sampling rate of 5.0 kHz) to measure the cardiomyocyte intracellular calcium concentration ([Ca^2+^]_i_)(5). Data were analysed offline as previously described(5).

**RNA sequencing sample preparation and analysis**

RNA was extracted using small [<200 nt] and large [>200 nt] nucleotide separation with the miRNeasy Mini Kit (Qiagen, UK) from BZ and RZ myocardial tissue from control Runx1^fl/fl^ and Runx1^Δ/Δ^ mice pre-MI as well as 1 day post-MI and whole LV myocardial tissue from Cbfβ^fl/fl^ and Cbfβ^Δ/Δ^ pre-MI and 7 days post-MI. Sequencing libraries were enriched using polyA tail selection and samples were run on a Next Seq500 Sequencing system (Illumina) at the Glasgow Polyomics research facility. The raw fastq files containing single-end 1 × 75 bp reads were pre-processed with Cutadapt (v.1.8) and Sickle (v.0.940) software (https://github.com/najoshi/sickle) to remove the 3' end adaptor and to trim the very low-quality reads, respectively. The quality threshold was set to 10 and no reads shorter than 54 bp were allowed to remain (sickle flags: -q 10, -l 54). The pre-processed reads were then aligned to the reference genome (Ensembl GRCm38.95) with Hisat264 (v2.1.0) and transcript expression quantification was performed using StringTie65 (v1.3.5). Gene level count matrices were generated with prepDE.py script as instructed in StringTie manual (http://www.ccb.jhu.edu/software/stringtie/index.shtml?t=manual) and differential expression were analysed using edgeR66 R-package to provide statistically significant gene-lists for a variety of between-groups comparisons. Global functional, network, and canonical pathway analyses were performed using Ingenuity Pathways Analysis (IPA). Gene lists and expression values were uploaded to Ingenuity® Pathway Analysis (IPA) software (QIAGEN Inc.)(6). Genes shown in the article text and figures were generated by IPA and therefore might exclude lncRNAs and pseudogenes that are not recognised by the IPA software. The core analysis feature was used to interpret the differentially expressed data (FDR ≤ 0.05), including biological (canonical) pathway analysis and an activity analysis z-score giving a pathway prediction of inhibition (blue) or activation (orange). Comparison analysis between specific groups were carried out to visualise relevant canonical pathways using right-tailed Fisher's Exact Test and corrected for multiple testing using Benjamini Hochberg.

**Electron Microscopy**

Hearts were perfused with cardioplegic solution, immediately followed by perfusion-fixation with iso-osmotic Karnovsky’s fixative (2.4% sodium cacodylate, 0.75% paraformaldehyde, 0.75% glutaraldehyde) and then separated into BZ and RZ. BZ and remote LV regions were then washed with 0.1 M sodium cacodylate, post-fixed in 1% OsO4 for 1 h, dehydrated in graded acetone, and embedded in Epon-Araldite resin as described before(7). All sections were placed on formvar-coated copper/palladium slot-grids, post stained with 2% aqueous uranyl acetate and Reynold’s lead citrate. Colloidal gold particles (15 nm) were added to both surfaces of the thick sections to serve as fiducial markers for tilt series alignment. Thin (90 nm) and semi-thick (300 nm, for dual-axis electron tomography) sections were prepared and imaged at the Electron Microscopy Core Facility, European Molecular Biology Laboratory (EMBL) Heidelberg, using 300 kV Tecnai TF30 (FEI Company, now Thermo-Fisher Scientific, Eindhoven, The Netherlands). For tomographic imaging, the specimen holder was tilted from +60° to −60° at 1° intervals. For dual-axis tilt series the specimen was then rotated by 90° in the X-Y plane, and another +60° to −60° tilt series was taken. The images from each tilt-series were aligned by fiducial marker tracking and back-projected to generate two single full-thickness reconstructed volumes (tomograms), which were then combined to generate a single high-resolution 3D reconstruction of the original partial cell volume. Tilt series were aligned, reconstructed, and combined using IMOD. Mitochondrial density, quantity, and damage was analysed using IMOD software and ImageJ. Mitochondrial density was calculated in each cardiomyocyte, represented as a percentage of the total cellular area. The number of mitochondria were also counted in the same cells, with mitochondrial size determined by dividing the total mitochondrial area by the number of mitochondria. Mitochondria were classified as damaged (e.g. Extended figure 6a and b) if: cristae and outer membrane integrity was disrupted (dissolved/damaged cristae); evidence of fusion of mitochondria with autophagocytic vesicles was present; or if there were mitophagosomes present. Damaged mitochondria were counted in each cell and the proportion present calculated by dividing the number of damaged mitochondria by the total. Mice were maintained under 1-1.5% of inhalational isoflurane and images taken using an ACUSON Sequola C512 system with an 18LS probe at 14 hz. All measurements were taken at the level of the papillary muscle to ensure uniformity between groups.

**Histology**

Mice were anaesthetised in an induction chamber at 4% isoflurane and once righting reflex was lost were sacrificed by cervical dislocation. Hearts were quickly removed, flushed with saline, and fixed in 10% buffered formalin for 24h before being embedded in a wax block for sectioning. Quantification of regional areas and infarct size was performed on Picrosirius Red-stained histological sections with ImageJ and Adobe Photoshop as previously described(1).

**Adenoviral knockdown of Runx1 in the border zone region**

The Ad-Runx1-shRNA(8) and a random scramble sequence (Ad-scramble-shRNA) were purchased from Vector Biolabs (USA; Gene ID: 12394). Ad-scramble-shRNA and Ad-Runx1-shRNA were validated in vitro using IP1B cells, a murine cell line with high Runx1 expression (obtained from the American Type Culture Collection; ATCC) (Extended Figure 7a and 7b). Following coronary artery ligation, the BZ area was injected with 5 x 10 μL of either Ad-scramble-shRNA or Ad-Runx1-shRNA (1 x10^9^ viral particles per heart) immediately following MI. Runx1 shRNA adenovirus encoded Runx1 shRNA short hairpin forward 5’-GATCCCCGGGCCCTCCTACCATCTATACTACTCGAGTAGTATAGATGGTAGGAGGGCTTTTTGG-3’ and reverse 5’- AATTCCAAAAAGCCCTCCTACCATCTATACTACTCGAGTAGTATAGATGGTAGGAGGGCCCGGG-3’. Scramble shRNA short hairpin contained forward: 5’- GATCCCCGGCAACAAGATGAAGAGCACCAACTCGAGTTGGTGCTCTTCATCTTGTTGTTTTTG-3’ and reverse 5’- AATTCAAAAACAACAAGATGAAGAGCACCAACTCGAGTTGGTGCTCTTCATCTTGTTGCCGGG-3’. We next determined the efficiency of transduction and *Runx1* expression (using RNAscope and Runx1 specific probes, as previously published^21^) in cardiomyocytes (identified by pericentriolar material 1; PCM-1 expression) and other cardiac cells. For each heart, positive (Ppib and Polr2a) and negative controls (bacterial dapB) were run (Extended Fig. 7c). There was no change in the total number of cardiomyocyte nuclei or non-cardiomyocyte nuclei between control and Ad-Runx1-shRNA groups within the IZ, BZ and RZ (Extended Fig. 7d-e). Hearts injected with the control Ad-scrambled-shRNA demonstrated the expected increase in Runx1 expression within the IZ and BZ region of the LV; whereas Runx1 expression within the RZ of the LV remained low (Extended Fig 8a and 8c). Injection of Ad-Runx1-shRNA into the BZ resulted in a 46% reduction in Runx1 expression within cardiomyocyte nuclei relative to Ad-scramble-shRNA injected hearts (39.1±4.5 vs. 72.3±3.1% of total number of cardiomyocytes, P<0.05; Extended Fig 8a, 8c). The reduction in Runx1 expression in cardiomyocytes was specific to the BZ region as there was no change in Runx1 expression within cardiomyocyte nuclei between the two groups in the other LV regions (Extended Fig 8a, 8c).

**AAV-mediated knockdown of Runx1**

Following coronary artery ligation, mice were intravenously injected via the tail vein with either AAV9-scramble-shRNA or AAV9-Runx1-shRNA (1x10^10^ virus particles per mouse) in a randomised fashion immediately following MI. shRNA sequences targeting murine Runx1 were generated using the short hairpin sequences in the adenoviral vectors for both Runx1 and scramble. Briefly, the complementary oligos of shRNA (Eurofins genomics, Germany) were annealed and cloned into a pZac2.1 vector, which was then used to produced recombinant AAV vectors in the AAV Vector Unit at IGGEB Triest, as described previously(9). Viral stocks were obtained by PEG precipitation and two subsequent CsCl_2_ Gradient centrifugations. Titration of AAV viral particles was performed by real-time PCR quantification of the number of packaged viral genomes as described previously(10). There was no change in the total number of cardiomyocyte nuclei or non-cardiomyocyte nuclei between control and AAV-Runx1-shRNA groups within the three regions assessed (Extended Fig. 7f and 7g). Using RNAscope, we determined the expression of Runx1 within AAV9-Runx1-shRNA injected MI hearts relative to AAV9-scramble-shRNA within RZ, BZ and IZ of each heart at day 7 post-MI. Runx1 expression within cardiomyocyte nuclei of the BZ of mice injected with AAV9-Runx1-shRNA was 65% of the levels observed in mice injected with AAV9-scramble-shRNA (35.0±3.6 vs. 53.9±6.4% of total number of cardiomyocytes; P<0.05; Extended Fig. 8b and 8d). There was no change in Runx1 expression within cardiomyocyte nuclei in the RZ of mice injected with AAV9-Runx1-shRNA relative to mice injected with AAV9-scramble-shRNA (14.3±2.3 vs. 20.1±2.8; P>0.05; Extended Fig. 8b and 8d). No significant change in Runx1 expression in cardiomyocytes was found in the IZ region (Extended Fig. 8b and 8d).

**Ro5-3335 knockdown of Runx1**

C57BL/6J mice were randomly assigned to receive either vehicle (100% DMSO) or 20 mg/ kg of Ro5-3335(11) (Tocris-Bioscience, UK) given subcutaneously. Mice assigned to protocol 1 received either Ro5-3335 or vehicle at 7, 5, 3 and 1 day before coronary artery ligation then again at 1, 3, 5 and 7 days following coronary artery ligation. Mice assigned to protocol 2 received Ro5-3335 or vehicle at the time of coronary artery ligation and on consecutive days until day 7.

**Ischaemia-reperfusion of ex vivo hearts**

Left intraventricular pressure was measured in Langendorff perfused hearts from adult male Wisar rats as previously described(12). Hearts were randomly assigned to receive either vehicle (1µM DMSO) or 1µM of Ro5-3335 after 27 min of global ischemia.  Infarct size was measured using triphenyltetrazolium chloride staining as previously described(12).

**Serum samples from humans with ST elevation MI (STEMI)**

A prospective single-centre cohort study was performed between January 2015 and September 2016 at a regional cardiac centre(12). Research staff screened patients with acute STEMI undergoing emergency invasive management. Systemic blood samples were taken at three time points: (i) pre-reperfusion of the occluded culprit coronary artery; (ii) 20 min post-primary percutaneous coronary intervention (PPCI); and (iii) at 24 h post-PPCI. Cardiac magnetic resonance imaging (MRI) was performed at 24 h and 6 months post-PPCI. The study protocol and consent processes were approved by the local Research Ethics Committee (REC 14/WS/0085). In all, 64 patients agreed to participate and provided written informed consent. The human studies conformed to the principles outlined in the Declaration of Helsinki.

**Serum samples from humans without MI (no MI population)**

Control samples from patients without MI originated from; (i) A single-centre prospective randomized cross-over study conducted between June 2016 and December 2016(13). Twenty healthy male smokers without a history of established cardiovascular, respiratory, or renal disease were included in the study. Samples and data used for the present research were from the first study visit. The study was approved by the University of Glasgow College of Medical, Veterinary and Life Sciences Research Ethics Committee (reference number 200150108) and complied with the Declaration of Helsinki. Informed written consent was obtained from all participants; and (ii) PRIORITY (EudraCT 20120-004523-4 and ClinicalTrials.gov NCT02040441) which was an investigator-initiated, prospective, double-blind, randomised, placebo-controlled, international, multicentre clinical and observational study in people with type 2 diabetes and normoalbuminuria(14,15). People aged 18–75 years with type 2 diabetes, preserved kidney function, and normo- albuminuria were recruited. Samples and data used for the present research were from the Glasgow biobank. The study was done in accordance with the International Conference on Harmonisation Good Clinical Practice guideline and the Declaration of Helsinki and approved by local Research Ethics Committees. All participants provided written informed consent.

Serum levels of Runx1 were measured by an enzyme-linked immunosorbent assay using the manufacturers recommended protocol (2B Scientific) and normalised to the total serum protein content using a bicinchoninic acid assay (BCA)(12).

**Statistics**

Data were expressed as mean ± SEM. Comparisons between two experimental groups were performed with the Student’s t-test on raw data before normalization to percentage change where appropriate. Comparisons between more than two groups were conducted on raw data with ANOVA. In experiments where multiple isolated cardiomyocyte observations (n) were obtained from each heart, we first ensured normality of the data distribution and then determined the differences between control and experimental mice using mean data from each heart (and not individual cardiomyocytes; IBM SPSS Statistics, version 22).

**References**

1. McCarroll CS, He W, Foote K et al. Runx1 Deficiency Protects Against Adverse Cardiac Remodeling After Myocardial Infarction. Circulation 2018;137:57-70.

2. Naoe Y, Setoguchi R, Akiyama K et al. Repression of interleukin-4 in T helper type 1 cells by Runx/Cbf beta binding to the Il4 silencer. J Exp Med 2007;204:1749-55.

3. Sohal DS, Nghiem, N., Crackower, M.A., Witt, S.A., Kimball, T.R., Tymitz, K.M., Penninger, J.M., Molkentin, J.D. Temporally regulated and tissue-specific gene manipulations in the adult and embryonic heart using a tamoxifen-inducible Cre protein. Circulation Research 2001;89:20-25.

4. van Duijvenboden K, de Bakker DEM, Man JCK et al. Conserved NPPB+ Border Zone Switches From MEF2- to AP-1-Driven Gene Program. Circulation 2019;140:864-879.

5. Elliott EB, Hasumi H, Otani N et al. K201 (JTV-519) alters the spatiotemporal properties of diastolic Ca(2+) release and the associated diastolic contraction during beta-adrenergic stimulation in rat ventricular cardiomyocytes. Basic Res Cardiol 2011;106:1009-22.

6. Kramer A, Green J, Pollard J, Jr., Tugendreich S. Causal analysis approaches in Ingenuity Pathway Analysis. Bioinformatics 2014;30:523-30.

7. Rog-Zielinska EA, Johnston CM, O'Toole ET, Morphew M, Hoenger A, Kohl P. Electron tomography of rabbit cardiomyocyte three-dimensional ultrastructure. Prog Biophys Mol Biol 2016;121:77-84.

8. Wang J, Wang X, Holz JD et al. Runx1 is critical for PTH-induced onset of mesenchymal progenitor cell chondrogenic differentiation. PLoS One 2013;8:e74255.

9. Ayuso E, Blouin V, Lock M et al. Manufacturing and characterization of a recombinant adeno-associated virus type 8 reference standard material. Hum Gene Ther 2014;25:977-87.

10. Arsic N, Zacchigna S, Zentilin L et al. Vascular endothelial growth factor stimulates skeletal muscle regeneration in vivo. Mol Ther 2004;10:844-54.

11. Jeong EM, Pereira M, So EY et al. Targeting RUNX1 as a novel treatment modality for pulmonary arterial hypertension. Cardiovasc Res 2022.

12. He W, McCarroll CS, Nather K et al. Inhibition of myocardial cathepsin-L release during reperfusion following myocardial infarction improves cardiac function and reduces infarct size. Cardiovasc Res 2022;118:1535-1547.

13. Kerr DMI, Brooksbank KJM, Taylor RG et al. Acute effects of electronic and tobacco cigarettes on vascular and respiratory function in healthy volunteers: a cross-over study. J Hypertens 2019;37:154-166.

14. Tofte N, Lindhardt M, Adamova K et al. Early detection of diabetic kidney disease by urinary proteomics and subsequent intervention with spironolactone to delay progression (PRIORITY): a prospective observational study and embedded randomised placebo-controlled trial. The Lancet Diabetes & Endocrinology 2020;8:301-312.

15. Lindhardt M, Persson F, Currie G et al. Proteomic prediction and Renin angiotensin aldosterone system Inhibition prevention Of early diabetic nephRopathy in TYpe 2 diabetic patients with normoalbuminuria (PRIORITY): essential study design and rationale of a randomised clinical multicentre trial. BMJ Open 2016;6:e010310.
